# Monitoring Respiratory Motion With Wi-Fi CSI: Characterizing Performance and the BreatheSmart Algorithm

**DOI:** 10.1109/access.2022.3230003

**Published:** 2022-12-22

**Authors:** SUSANNA MOSLEH, JASON B. CODER, CHRISTOPHER G. SCULLY, KEITH FORSYTH, MOHAMAD OMAR AL KALAA

**Affiliations:** 1 Spectrum Technology and Research Division, Communications Technology Laboratory, National Institute of Standards and Technology, Boulder, CO 80305, USA; 2 Office of Science and Engineering Laboratories, Center for Devices and Radiological Health, U.S. Food and Drug Administration, Silver Spring, MD 20993, USA

**Keywords:** Channel state information, deep learning, LSTM, MIMO-OFDM, respiration monitoring, respiratory motion classification, Wi-Fi

## Abstract

Respiratory motion (i.e., motion pattern and rate) can provide valuable information for many medical situations. This information may help in the diagnosis of different health disorders and diseases. Wi-Fi-based respiratory monitoring schemes utilizing commercial off-the-shelf (COTS) devices can provide contactless, low-cost, simple, and scalable respiratory monitoring without requiring specialized hardware. Despite intense research efforts, an in-depth investigation on how to evaluate this type of technology is missing. We demonstrated and assessed the feasibility of monitoring and extracting human respiratory motion from Wi-Fi channel state information (CSI) data. This demonstration involves implementing an end-to-end system for a COTS-based hardware platform, control software, data acquisition, and a proposed processing algorithm. The processing algorithm is a novel deep-learning-based approach that exploits small changes in both CSI amplitude and phase information to learn high-level abstractions of breathing-induced chest movements and to reveal the unique characteristics of their difference. We also conducted extensive laboratory experiments demonstrating an assessment technique that can be replicated when quantifying the performance of similar systems. The results indicate that the proposed scheme can classify respiratory patterns and rates with an accuracy of 99.54% and 98.69%, respectively, in moderately degraded RF channels. Comprehensive data acquisition revealed the capability of the proposed system in detecting and classifying respiratory motions. Understanding the feasible limits and potential failure factors of Wi-Fi CSI-based respiratory monitoring scheme — and how to evaluate them — is an essential step toward the practical deployment of this technology. This study discusses ideas for further expansion of this technology.

## I. INTRODUCTION

Information about how a patient is breathing (e.g., respiratory rate (RR) and pattern) can be valuable in many medical situations. This information can be used to detect a variety of respiratory-centric conditions like sleep apnea, asthma, anxiety, pneumonia, cardiovascular diseases, and chronic obstructive pulmonary diseases [1].

Traditional respiratory monitoring methods, such as respiratory airflow, respiratory sounds, and chest wall movements, are contact-based and require a physical connection between the patient and a monitor. This connection to a monitor commonly requires the patient to be present in a medical office or a hospital– a criterion that may be difficult to meet for the elderly, disabled, or patients in a telehealth setting. Moreover, some patients might not be comfortable with wearing a monitoring device. These limitations and discomfort can influence the monitoring results by causing changes in respiratory motion and rate, possibly leading to inaccurate respiratory information.

Contact-less monitoring systems have been developed to overcome some of the drawbacks of traditional respiratory monitoring methods. Non-contact-based monitors untether the patient while aiming to provide accurate monitoring of respiratory activity. For instance, optical camera-based methods are presented in [2] and [3], in which the RR is measured based on the breathing signals extracted from the video streams. In this method, the RR accuracy strongly depends on the availability of a line-of-sight path between the patient and the monitor as well as ambient lighting conditions. In another effort, an audio-based method was proposed in [4] in which the RR measurement was based on the human breathing sound captured by a smartphone’s microphone. The accuracy of this method is sensitive to the background noise and depends upon the sensing distance.

To mitigate these limitations, radio frequency (RF)-based RR estimation techniques– such as Doppler radar [5], [6], Ultra-wideband (UWB) radar [7], [8], and frequency-modulated continuous-wave (FMCW) radar [9], [10], [11]– have been proposed. Utilizing the propagation of RF electromagnetic waves, these methods can sense chest movement, even in a complicated indoor environment. The main idea behind these methods is that the human’s breath-induced inhalation and exhalation (i.e., chest movement) can modulate the amplitude and phase of a signal propagating from a transmitter to a receiver. Upon reception of the signal at the receiver, the RR can be measured based on the changes in the received signal that occurred as it passed through the propagation channel.

Wi-Fi-based respiratory monitoring schemes have the advantage of utilizing commercially available off-the-shelf (COTS) devices and are a subset of RF-based sensing techniques that can overcome the challenges of cost, complexity, specialized hardware, and scalability. These respiratory monitoring schemes utilize existing Wi-Fi signals and hardware (e.g., access points and client devices) that have become ubiquitous in daily life. Among these schemes, the use of the Received Signal Strength Indicator (RSSI) has gained popularity on account of its accessibility on most Wi-Fi devices [12], [13], [14], [15]. RSSI provides the power measurement of the received signal. It is shown that changes in the RSS can be used for detecting some changes in the channel conditions. For example, Patwari et.al. [12] and [13] detected the breathing rate based on the extracted RSS measurements from wireless sensor nodes. This method, however, needs additional wireless network infrastructure as well as the high-density placement of sensor nodes. Authors in [14] proposed a method, called *UbiBreathe*, that utilizes RSSI for estimating the breathing rate. *UbiBreathe* accurately estimated the RR if the Wi-Fi devices were held close to the patient’s chest or placed in the line-of-sight path between the transmitter and the receiver. In another instance, to estimate heart and breathing rates, authors in [15] proposed a method called *mmVital*, that exploits the RSS of 60 GHz millimeter-wave signals. Although RSS is available and accessible on many Wi-Fi devices, its susceptibility to the channel noise imposes a ceiling on the level of accuracy in a RR estimate [16].

In addition to RSS, Wi-Fi channel state information (CSI) can be another source of useful information in Wi-Fi networks. Whereas RSS reports how much a signal is attenuated, CSI signals are typically used to delineate how a signal propagates from a transmitter to a receiver in a multi-path environment. CSI signals are available as complex values, embedding information about changes to the propagating signal in terms of its magnitude and phase. Therefore, CSI-based methods can outperform RSS-based methods because of the fine signal granularity and additional information provided by the phase.

The CSI mechanism was designed to “sound” the propagation channel [17]. The information on how the channel attenuates and delays a signal is then used by the Wi-Fi access point (AP) to optimize its link with the client. By design, the CSI signal is sensitive to the movement of objects; a feature that can be utilized for sensing purposes such as indoor localization/tracking [18], [19] and activity/gesture recognition [20], [21], [22].

The use of Wi-Fi CSI is an emerging method for estimating respiratory activity. Observing the variations of the CSI as it is impacted by breathing-induced chest movement facilitates estimating the breathing rate and tracking the breathing pattern. Moreover, unlike radar-based RR estimation methods, the CSI-based method has the potential to be widely deployed while performing short- or long-term respiratory monitoring using existing Wi-Fi hardware. A detailed discussion of the prior work in using Wi-Fi CSI for respiratory monitoring can be found in Section II.

In this paper, we show the results of an in-depth investigation on using Wi-Fi CSI to estimate respiratory patterns and rates. Although other investigations of Wi-Fi CSI exist, previous studies have relied on experimental platforms or were limited to specific scenarios. For instance, one of the main limitations of the proposed methods in [23] and [24] is that they only utilized data collected from two different volunteers in different sleeping postures, one for establishing the database and the other for the testing data. Therefore, the generality of their system for different sleepers has yet to be investigated thoroughly. As another example, in [25], the experiments are conducted with six participants over three months to generate a more enhanced data set while the participants lay in a bed and controlled their breathing rates to generate a range of 12 to 18 breaths per minute respiratory motion. Such algorithm training systems incur high costs (in terms of time and complexity) to enroll sufficient human subjects under various conditions to generate databases for training that are rich in the factors that could affect performance and support the development of robust and generalizable algorithms. In this work, we integrate a system capable of extracting the human respiratory motion and rate from Wi-Fi CSI data in a variety of scenarios by considering a diverse range of respiratory rates, respiratory patterns, environments with different attenuations, data acquisition lengths, and Wi-Fi frame rates. This system consists of a COTS-based hardware platform, control software, and a proposed processing algorithm. The processing algorithm is a novel deep-learning-based respiratory monitoring approach that exploits both the amplitude and phase information of Wi-Fi CSI signals to reveal unique characteristics of different minute movements and to learn high-level abstractions of breathing-induced chest movements–leading to an accurate respiratory pattern and rate estimation. Beyond evaluating the specific processing algorithm presented here, the method used for the evaluation is extensible to other, similar technologies. This platform and processing algorithm allow for a deeper understanding of technologies using Wi-Fi CSI for respiratory estimation and monitoring, their feasible limits, and potential failure factors. This information is valuable to medical device designers and regulators when considering this technology in addition to its utility in designing experiments for other types of CSI-based monitoring systems.

### A. CONTRIBUTIONS

**FIGURE 1.**
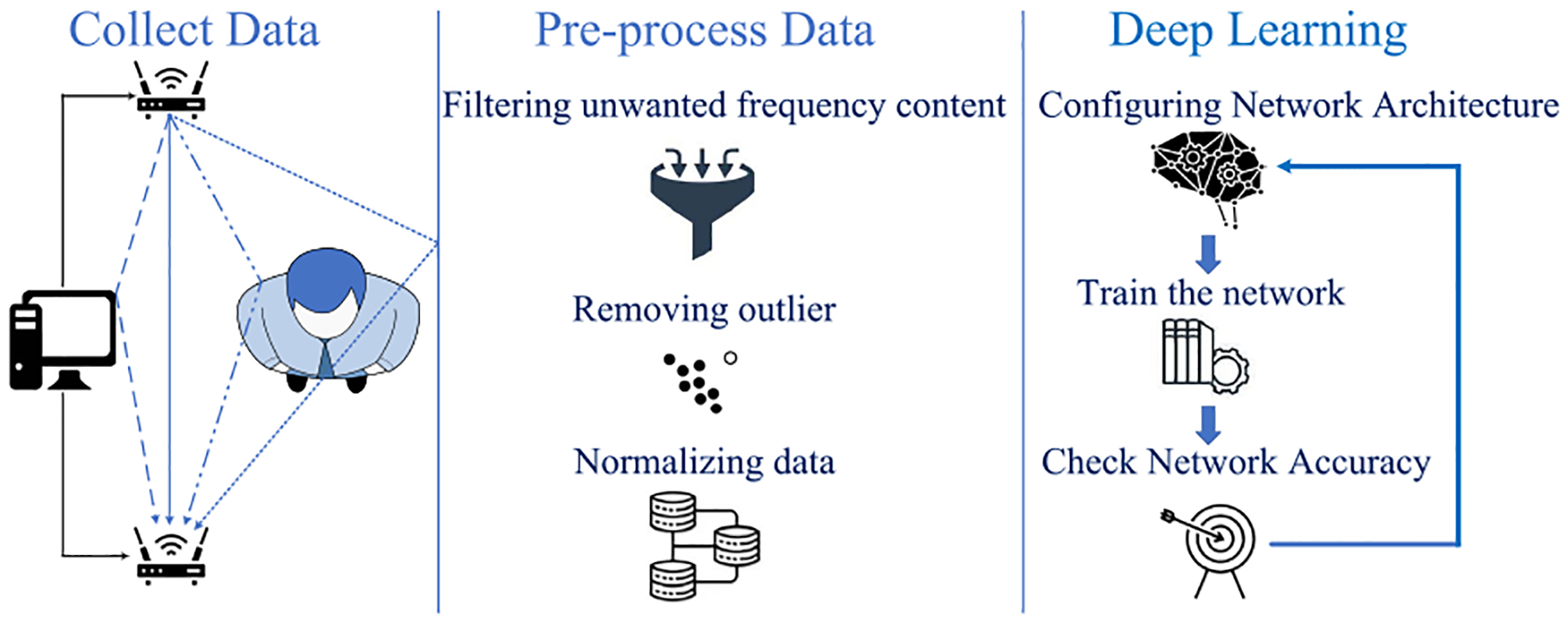
Architecture of the proposed system.

This work differs from previous studies by detailing the end-to-end implementation, functional description, and characterization of a Wi-Fi CSI-based respiratory monitoring system. Figure 1 provides a high-level overview of the proposed system. The technical contributions of this work can be summarized as:

- Demonstration and assessment of the feasibility of the monitoring human respiratory motion and rate by use of the Wi-Fi CSI parameters. This demonstration includes the implementation of an end-to-end system (i.e., hardware, firmware, data acquisition, and signal processing).
- Novel machine-learning algorithm that leverages deep learning techniques to extract the respiratory pattern and rate from the raw CSI data set; separating the motion from the surrounding environment and noise.
- A series of laboratory experiments and metrics that could be used to explore the performance of any technique and algorithm in this domain. The technique developed here is used as an example. Moreover, measurements are described in a general nature, enabling them to be replicated when quantifying the performance of similar systems.

### B. PAPER ORGANIZATION AND NOTATION

The remainder of this paper is organized as follows. Section II provides background on Wi-Fi sensing and respiratory rate estimation as well as a review of the related work. In Section III, we describe the CSI model and introduce the main assumptions for the analysis conducted in this paper. Section IV presents the concept of the Long Short Term Memory (LSTM)-based method and demonstrates the design of the introduced RR and pattern classification scheme for multiple-input multiple-output orthogonal frequency-division multiplexing (MIMO-OFDM) systems. Experiment setup and classifier performance assessment are discussed in Section V and Section VI, respectively. Finally, the conclusion and future work are presented in Section VII.

#### Notation:

Throughout the paper, normal letters are used for scalars. Boldface capital and lower case letters denote matrices and vectors, respectively. The sets of complex numbers is denoted by ℂ. All vectors, e.g., $\text{a}\in {\mathbb{C}}^{K}$, are assumed to be tall (column) vectors with *K* entries. The transpose of matrix **A** is denoted by **A**^*T*^ . The angle is symbolized by ∠ and |**a**| returns the complex magnitude of each element in the complex vector **a**. Re(**a**) and Imag(**a**) denote the real and imaginary part of the complex vector **a**, respectively. The *i*-th element of vector **a** is denoted by **a**_*i*_. We define sets with calligraphic letters, i.e., 𝒦, and the cardinality of a set is denoted by |𝒦|. The element-wise product of two functions is indicated by ⊙. Finally, exp and tanh indicate the standard exponential function and hyperbolic tangent function, respectively.

## II. PREVIOUS USE OF CSI FOR SENSING

This section presents a comprehensive overview of prior work that utilizes Wi-Fi CSI for human respiratory detection and quantification. This overview is presented to show the evolution of this technique and illustrate where advancements may be possible.

In general, previous work on CSI-based respiratory monitoring methods can be divided into two categories: (i) pattern-based methods and (ii) model-based methods.

Pattern-based CSI respiratory monitoring methods estimate the RR based on the changes in the CSI patterns. Among these methods, the power spectral density (PSD) method and the inter-breath interval (IBI) method, also known as the peak detection method, are the most commonly used. RR estimated by the former method is equal to the frequency in which the PSD is maximized while the rate estimated by the latter is the inverse of the average time differences between the peaks of the selected stream. Liu, et al. [23] began propounding the idea of pattern-based CSI breathing monitoring. They proposed a method, called *Wi-sleep*, that utilizes Wi-Fi CSI amplitude data for monitoring human respiration during sleep. In [25], the authors utilized CSI amplitude to estimate both breathing and heart rates. However, that specific method measures RR when the person is asleep and requires the person to remain asleep. The results of this study were extended in [24] in which vital signs were inspected in various sleeping postures. The above-mentioned approaches utilize a periodogram to analyze the spectrum. The disadvantage of this method is that it may take a long time to accurately estimate RR. In [26], a new method, called *DeMan*, was proposed to estimate RR while the subject is in a seated position. This method looks into the probability of a measured signal having the same flow rate as human breathing. The chest movement– and thus RR– will be detected if the measured signal frequency falls within the range of human breathing frequencies. While these methods each utilize the CSI amplitude, *PhaseBeat* [27] and *TensorBeat*, [28] exploited the CSI phase difference between two antennas. These works utilized phase data for monitoring the breathing signals of people. However, their performance is inconsistent due to the lack of theoretical justification [29].

Although the above-mentioned methods experimentally reveal that utilizing Wi-Fi CSI provides respiration rate, these experiments have only been done and proven during short-duration measurement campaigns in ideal scenarios and controlled settings that make it difficult to replicate. Therefore, the work described in this paper attempts to perform respiratory pattern and rate monitoring using Wi-Fi CSI without these idealities by conducting extensive measurements that could be replicated when quantifying the performance of similar systems. Moreover, we exploit both CSI amplitude and phase information in an attempt to provide an accurate estimation of respiratory motion and rate. The proposed method for extracting the respiratory motion and rate from CSI data includes a theoretical discussion to illustrate *how* and *why* the proposed method works. This may aid others in developing similar methods and help identify CSI-based sensing technologies’ limits..

In previous studies, the theoretical foundation of the principle of pattern-based methods is missing. To overcome this gap, model-based methods have been developed. These models estimate RR by relating breathing to the received CSI measurements using statistical models. A theoretical model for estimating the breathing rate based on the Wi-Fi signals in the Fresnel region^1^ was presented in [30]. In this study, radio propagation is utilized to investigate any diffraction loss due to obstruction between transmitter and receiver, and the main factors –such as user location and body orientation– that affect the detectability of the breathing signal were verified. This study also showed that the RR could not be effectively estimated in certain spatial regions (e.g., the area around the Fresnel Zone). To improve the estimation accuracy, *TinySense* was proposed in [31]. This method utilizes multiple-input multiple-output (MIMO) technology to estimate RR based on the sub-carriers influenced by the multi-path effect. Another study by Zhang et al. [32] showed that any movements in the Fresnel zone could significantly affect the amplitude and phase of the received signal. Zeng et al. in [33] proposed a method, called *FullBreath*, to estimate the RR by leveraging the CSI phase and amplitude data that utilize the changes of CSI at partial breathing depth. Even though this method solves the blind-spot problem at some breathing depths, there are still situations in which the CSI cannot be detected effectively. To extend the detection range, authors in [34] proposed a method, called *FarSense*, in which the ratio of CSI readings of two antennas is utilized as the RR sense metric. The line of this work is further expanded in [35] in which *ResBeat* was proposed to overcome the poor respiratory detection problem by utilizing the bimodal CSI data. However, these remain model-based methods; reports of their performance in physical tests is not present in the scientific literature.

One hurdle to applying these methods to arbitrary environments is the high computational cost of the calculations involved in estimating the RR. This high computational cost could limit the usable scenarios to those where sufficient computing power (or time) is available.

In recent years, machine learning (ML) innovations have been transformative in many industries. Wireless communications are also poised to benefit from these advances. For example, ML algorithms such as *k* Nearest Neighbor (kNN), Support Vector Machine (SVM), and Self-Organizing Map (SOM), are widely used for detection, recognition, and multi-class classification applications. ML has been applied to Wi-Fi RSS applications in [16] and [36] and to Wi-Fi CSI relevance in [37] in the domain of gesture sensing. Specifically, in [16], the authors apply an ML algorithm to the *real-value* RSS to detect human hand motions around a user device. Also employing the *real-value* RSS, authors in [36] developed a human presence detection technique to support energy saving by automating appliances. [37] applied an ML algorithm on the *amplitude* of the Wi-Fi CSI data in the domain of identifying human gestures and activities. In contrast, our proposed learning scheme considers the *complex* CSI values, i.e., both CSI amplitude and phase information, and not only classified breathing rates but also classified breathing patterns which are a function of user gesture. Some of these proposed algorithms achieved an accuracy of over 75% for identifying all activities like walking, running, standing up, sitting down, and lying down.

These results make the use of ML a prime candidate for detecting respiratory activity. In this article, we propose a suite of algorithms using innovations in ML for assessing and improving the accuracy of respiratory rate and pattern classification. We treat a respiratory motion classifier scheme’s mapping function from the input to the output variables as an unknown non-linear function. Deep learning techniques are used to approximate this mapping function. If the mapping can be learned with high accuracy/performance by a moderately-sized deep learning network, the computational costs of estimating respiratory patterns and rates can be reduced significantly– leading to an accurate estimation of respiratory motions in real-time with already deployed hardware and infrastructure. Only a software update would be required to acquire and process data directly on the COTS Wi-Fi hardware.

Deep learning techniques, which include deep neural networks such as fully connected neural networks that are defined by their multi-layer perceptrons (MLPs) [38], convolutional neural networks (CNNs), and recurrent neural networks (RNNs), are advantageous in analyzing and interpreting vast amounts of data as they make the learning process faster and easier. In CNNs, layers are sparsely connected rather than fully connected– leading to an easier and more effective learning process than MLPs. Whereas in MLPs/CNNs data flows straight between layers without going backward, in RNNs information is cycled through a loop– flows back into the current node (also known as recurrent connections). By doing that, RNNs utilize their memory to process a sequence of inputs, making it applicable to sensing and prediction tasks. However, these techniques suffer from the effects of exploding/vanishing gradient that makes the model unstable and unable to learn long-term dependency from training data [39].

To overcome this limitation, Long Short-Term Memory (LSTM) networks have been proposed [40]. LSTM is a type of RNN that introduces a new structure, known as a memory cell, with additional “forget” gates compared to simple RNNs. LSTM has a global state which is maintained among all the inputs effectively transferring the input’s context to all future inputs using this state. This new structure is capable of learning/capturing long-term dependencies between time steps of data without suffering from vanishing and exploding gradient problems, recognizing patterns in data sequences, and classifying sequential data– which makes it applicable to processing long time series sequences such as CSI data. Therefore, in Section IV we describe an LSTM-based algorithm to estimate respiratory pattern motion and rate.

## III. CHANNEL STATE INFORMATION MODEL

While Wi-Fi networks are primarily used to provide internet access and connect local area networks, they have substantial potential to sense the environment and changes caused by the human body’s movements. In the case of sensing respiratory motion and rate with Wi-Fi CSI, the human body slightly perturbs the Wi-Fi’s propagation environment as the human chest expands and contracts during the breathing process. These small perturbations caused by the chest can be detected as small changes in Wi-Fi CSI data.

Wi-Fi devices that follow the IEEE 802.11n/g standard often feature multiple transmit and receive antennas for MIMO communications and embrace OFDM in which each Wi-Fi frame is transmitted over one of up to 56 sub-carriers when transmitting with 20 MHz bandwidth or up to 114 sub-carriers when transmitting with 40 MHz bandwidth. The experiments designed in this work utilize Wi-Fi routers to send/receive Wi-Fi traffic with the CSI signal. These routers operate on the 5 GHz Industrial, Scientific, and Medical (ISM) unlicensed frequency band. According to the IEEE 802.11 standards, for the Wi-Fi routers operating on the 5 GHz frequency band, channels are numbered at 5 MHz spacing within a band, and a number linearly relates to the channel’s center frequency. For our application, the center frequency of the Wi-Fi routers is set to 5280 MHz, with frequency ranges of 5270 − 5290 MHz (20 MHz bandwidth), transmitting over 56 sub-carriers with unique channel propagation characteristics. Unlike available studies in the literature, such as [23], [24], and [25], that select the most appropriate subcarriers for tracking respiration, we assume that each subcarrier contains as much information about breathing as others and thus consider all 56 subcarriers in the channel. All CSI streams are used to develop an accurate estimate of the respiratory motion regardless of the frequency-dependent propagation effects between the sub-carriers. Our processing algorithm examines the difference between CSI signals at a single subcarrier in time. As long as the propagation effects are constant over that small time interval (on the order of hundreds of mS), their impact is insignificant because we are only interested in the relative change. The frequency diversity of these sub-carriers brings about different scattering as the human chest moves, resulting in different CSI amplitudes and phases at each subcarrier. The information within the changing CSI amplitude and phase values hold the potential for capturing the minute movements, leading to a more precise and accurate breathing rate and pattern detection. In reflective or non-line-of-sight environments, how each sub-carrier fades and reflects through the environment can create additional opportunities to capture and resolve the respiratory pattern motion and rate. Therefore, analyzing the Wi-Fi CSI data stream– which refers to the time series of CSI values associated with transmit antenna *n*_*t*_, receive antenna *n*_*r*_, and sub-carrier *c*– provides an opportunity to estimate respiratory motion.

The raw Wi-Fi CSI data can be read from modified device drivers for several COTS Wi-Fi network interface cards (NICs) or access points, such as Intel Wi-Fi Link 5300 NIC [41] and the Atheros AR9580 chipset [42]. Modified device drivers output the raw CSI data so it can be processed in other algorithms, such as the one discussed in Section IV.

In wireless communication systems, the transmitted signal undergoes degradation during propagation through the wireless channel, including attenuation as the signal travels through space or in the presence of physical objects. As the signal degrades, it often reflects, diffracts, and scatters. CSI delineates how a signal propagates from a transmitter (Wi-Fi AP) to the receiver (Wi-Fi client) in a multi-path environment and provides information regarding the various effects, such as time delay, amplitude attenuation, and phase shift on each Wi-Fi signal.

The received signal, in a narrow-band, flat fading OFDM channel, can be modeled as

(1) y(f,t)=H(f,t)x(f,t)+n(f,t),

where **y**(*f*, *t*), **H**(*f*, *t*), **x**(*f*, *t*), and **n**(*f*, *t*) denote the received signal, the channel response matrix, the transmitted signal, and the additive white Gaussian noise, respectively, for any carrier frequency *f* ∈ ℱ and any instant *t* ∈ 𝒯, where ℱ and 𝒯 are the sets of all sub-carriers and time instants, respectively. The channel matrix **H**(*f*, *t*) reflects the channel information at the sub-carrier level, explaining how multi-path effects impact CSI amplitude and phase and how signals of different frequencies are affected by the same channel. Each CSI entry of matrix **H** can be represented as

(2) $$h(f,t)=\sum_{\ell =1}^{L}\xi_{\ell }(t)\text{ exp }\left(-j2\pi f\tau_{\ell }(t)\right),$$

where *L* is the total number of multi-path components, and *ξ*_*ℓ*_(*t*) and *τ*_*ℓ*_(*t*) denote the complex gain and the propagation delay of the *ℓ*-th multi-path component at instant *t*, respectively. Note that, in the absence of dynamics in the environment, the complex gains *ξ*_*ℓ*_ and delays *τ*_*ℓ*_ are time-invariant. However, where there are physical changes in the environment, such as movements of the transmitter, receiver, or surrounding objects and humans, the CSI amplitude and phase will be affected and become time-varying.

**FIGURE 2.**
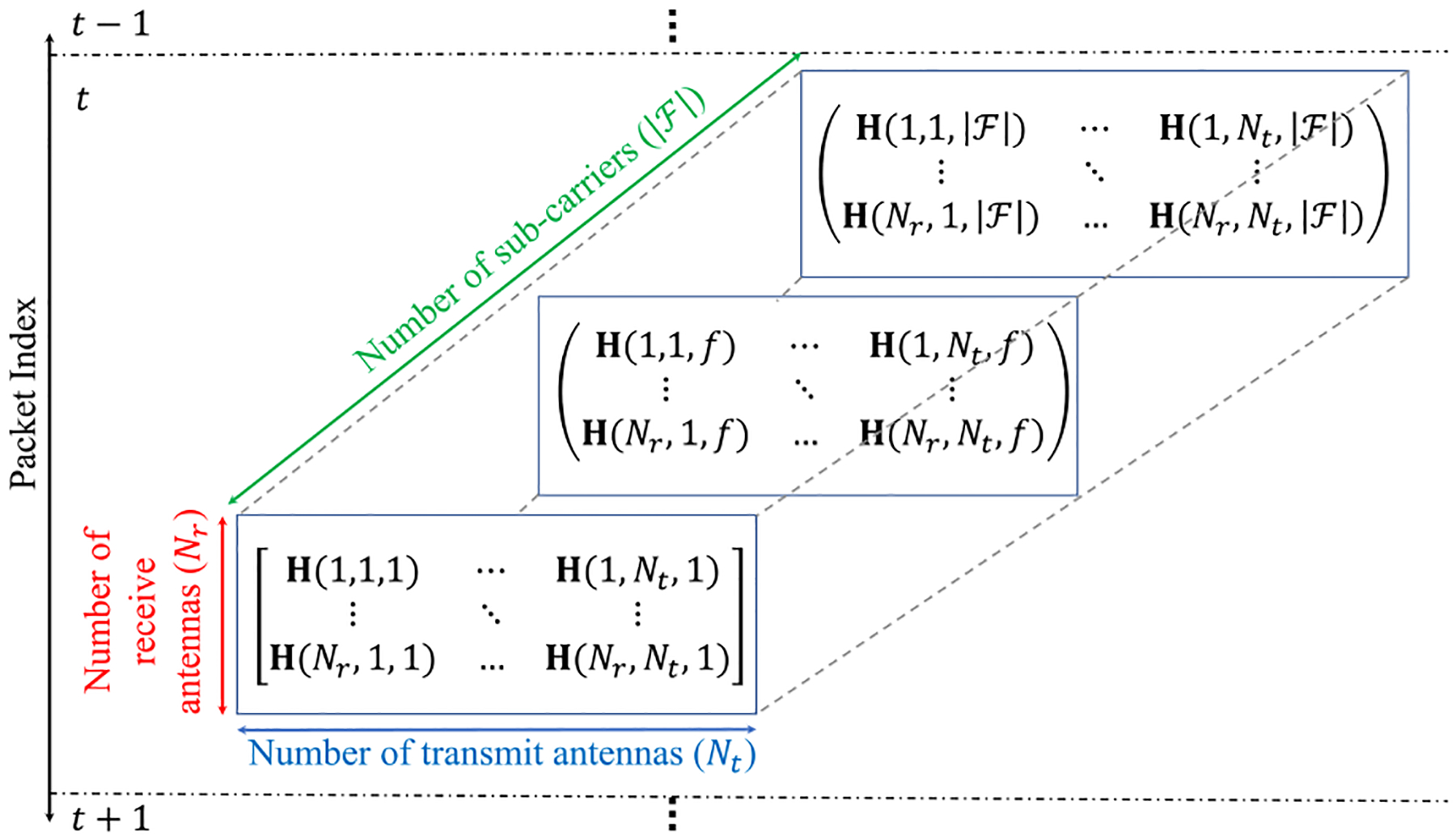
4D CSI tensor: A time series representative of CSI matrices for a MIMO-OFDM wireless network.

For a Wi-Fi MIMO-OFDM channel in which the transmitter and receiver are occupied with *N*_*t*_ transmit antennas and *N*_*r*_ receive antennas, respectively, and the total number of sub-carriers is |ℱ|, the CSI matrix is a 3D complex matrix $\text{H}\in {\mathbb{C}}^{N_{r}\times N_{t}\times \left|ℱ\right|}$ representing amplitude attenuation and phase shift of multi-path channels. To be specific, assume the Wi-Fi access points support transmission over a single 20 MHz channel with 3 transmit and 3 receive antennas. For the frame received over the time window 𝒯, the receiver observes a maximum of 3 × 3 × 56 complex CSI pairs, i.e.,

(3) $$\text{H}=\left[{\text{h}}_{1},\;\ldots \;,{\text{h}}_{m},\;\ldots \;,{\text{h}}_{504}\right],$$

where ${\text{h}}_{m}\in {\mathbb{C}}^{T}$ is the *m*-th stream of the Wi-Fi CSI data and presents both amplitude attenuation and phase shift impacted by a multi-path environment. It can be represented as

(4) $${\text{h}}_{m}=\left|{\text{h}}_{m}\right|\text{ exp }\left(j∠{\text{h}}_{m}\right),$$

where |**h**_*m*_| denotes the amplitude and represents the strength of the Wi-Fi signal, and ∠**h**_*m*_ indicates the phase values of the *m*-th CSI data stream and signifies the periodic variation of the signal with the propagation distance. Therefore, as demonstrated in Figure 2, the CSI can also be presented as a 4D CSI tensor, i.e., $\text{H}\in {\mathbb{C}}^{N_{r}\times N_{t}\times |ℱ|\times |𝒯|}$, providing additional information in the time domain.

### A. DATA GENERATION

For each respiratory motion pattern/rate, the complex Wi-Fi CSI data streams **h**_*m*_, *m* ∈ {1, …, 504}, and their corresponding motion-related value are collected for a Wi-Fi frame rate of 10 and for *T* = 60 seconds through the Wi-Fi devices used in our experiment (See Section V for the details of our experimental setup). Once collected, these CSI data streams and their corresponding respiratory motion pattern/rate values are split into a training set, 𝒩_training_, and a test data set, 𝒩_test_ (See Sections IV and VI for a detailed explanation). The 2-tuples ({**h**_*n*_},{RR_*n*_})_*n*∈𝒩_ or ({**h**_*n*_},{Pattern_*n*_})_*n*∈𝒩_ is referred to the *n*-th training/test samples where 𝒩 = 𝒩_training_∪𝒩_test_ denotes the set of all measured complex value Wi-Fi CSI data streams indices. Training data set will be used to develop the model, while predictions will be made on the test data set.

### B. DATA PRE-PROCESSING

**FIGURE 3.**
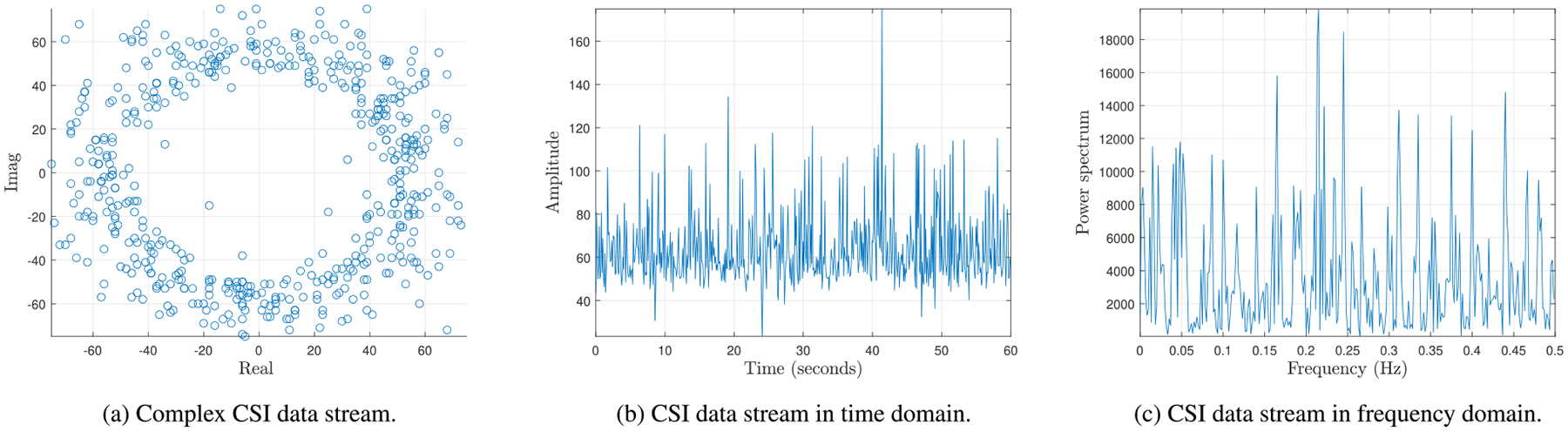
FFT-based spectrum pre-processing on a CSI data stream of a normal breathing pattern with RR = 15 BPM.

Before feeding the raw CSI data streams to our deep neural network, we first pre-process the data. Even though CSI is independent of the channel noise, it is prone to changes caused by the fluctuations in transmission rate, transmission power, and the internal CSI reference level of the Wi-Fi network cards [43]. These changes or measurement noises are the sources of high-frequency artifacts and outliers in CSI signals. Our method starts with data pre-processing – which includes removing outliers and unwanted frequency content from CSI signals. It has been shown that applying low pass filters to the CSI *amplitude* can effectively remove/mitigate the high-frequency noise [20], [44] while making use of Hample filters on the CSI *amplitude* can help alleviate outliers [45]. However, in this work, we exploit the complex CSI values,^2^ i.e., both CSI amplitude and phase information. The expected inputs of the low pass and Hample filters should be real, and applying these filters on the complex CSI data stream means performing filtering operating on the magnitude and phase components separately. Results show that applying a low pass filter on the magnitude and phase components of the CSI data stream separately and then passing these outputs of the low pass filter into the Hample filter does not provide high performance, especially in the high attenuated scenario where the useful signal is attenuated and embedded in noise. Plotting the CSI signals shows that identifying the frequency components of the signal in these scenarios is difficult. Since the effectiveness of the use of frequency analysis-based methods for estimating the respiratory motion-related values has been extensively demonstrated in previous works, we apply a fast Fourier transform (FFT)-based spectrum pre-processing algorithm, as depicted in Figure 3, that transforms the CSI data stream to the frequency domain, identifies the breathing frequency from a power spectrum, and extracts and learns respiratory features in the CSI signal. Next, all features are normalized to help ensure that the randomly initialized weight matrices correspond to the feature scale. Feeding different features with widely different scales to the learning model will cause the network to weigh the features unequally, which causes a false prioritization of some features over others and prevent the network from effectively learning the problem. Moreover, normalization may speed up learning and improve network convergence, leading to lower training times. We take the standard approach that scales the inputs to have zero mean and a standard deviation of one.

## IV. BREATHESMART ALGORITHM

Our proposed processing algorithm, called *BreatheSmart* hereafter, is a novel deep-learning-based respiratory monitoring approach that examines both the amplitude and phase of the Wi-Fi CSI data to reveal unique characteristics of small movements and to learn high-level abstractions of breathing-induced chest movements–leading to an accurate and robust estimation of respiratory motion. As discussed in SectionII, several different algorithms can be applied to CSI data for this purpose. In keeping with the theme of this work, this algorithm can be viewed as both advancing the state-of-the-art and as a springboard for future development. The detailed derivation shown here enables the detailed assessment of performance shown in Section VI.

The main idea is to treat the CSI-based respiratory monitoring algorithm as a *black box* and to implement a deep neural network that can learn the input-output relation of this *black box*, i.e.,

(5) y=f(x),

where **x** is the complex value Wi-Fi CSI data stream, *y* denotes the respiratory motion (either respiratory pattern or rate) that corresponds to the Wi-Fi CSI data stream **x**, and *f*(·) is a non-linear mapping function. This mapping function is learned using training data and then utilized later by predicting the respiratory motion associated with new (i.e., test) Wi-Fi CSI measurement data. With different respiratory rates and patterns, this problem is often framed as a multi-class classification problem and implemented as predicting the probability of the Wi-Fi CSI data stream belonging to each known respiratory motion pattern/value that is mutually exclusive.

### A. NETWORK ARCHITECTURE

**FIGURE 4.**
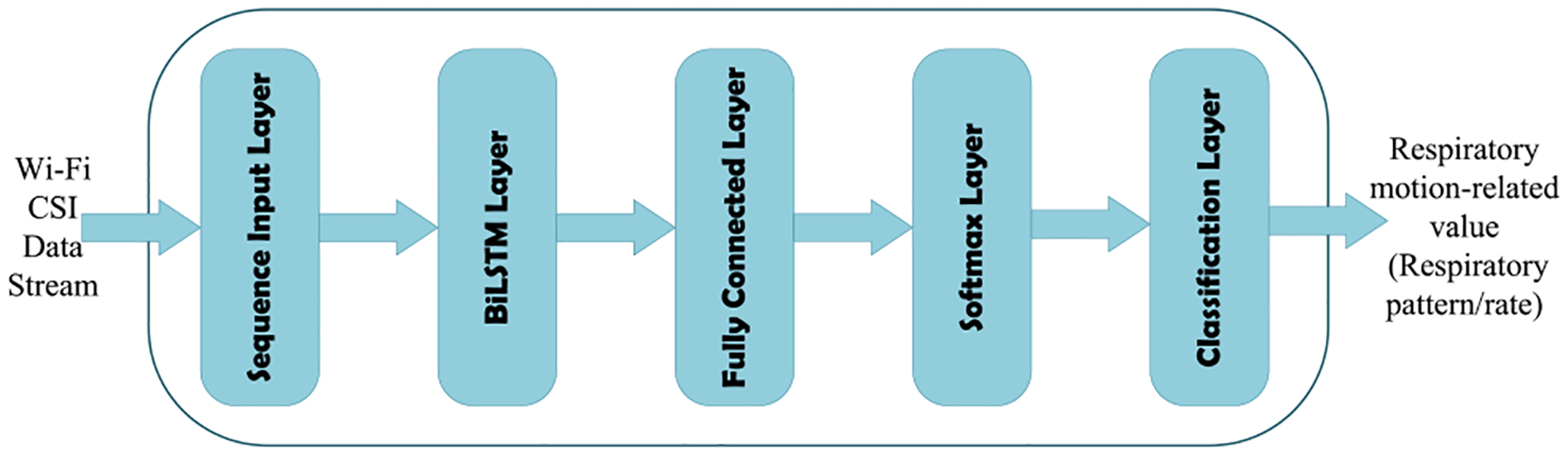
A high abstraction of LSTM network architecture used in this paper.

As depicted in Figure 4, our proposed algorithm uses an LSTM network. Its core components are a sequence input layer and a bidirectional LSTM (BiLSTM) layer. While the sequence input layer feeds time-series Wi-Fi CSI data stream **h**_*n*_ to the network, the BiLSTM layer learns long-term bidirectional dependencies between time steps of **h**_*n*_ over time. However, the BiLSTM layer’s output is not comparable to our respiratory motion-related values. To deal with this issue, we add a fully connected layer on top of the BiLSTM layer that combines all the information learned by the BiLSTM layer and uses them to classify the respiratory motion-related values. The output of the fully connected layer then passes through a softmax layer. This layer converts the output of the fully connected layer into probabilities– each probability assigns to a respiratory motion-related value/class. The last layer of the proposed network is a classification layer that takes the outputs of the softmax layer and assigns each Wi-Fi CSI data stream **h**_*n*_ to one of the respiratory pattern/rate classes (See Appendix A for a detailed explanation).

### B. TRAINING AND TESTING STAGES

In the training stage, the *BreatheSmart* network is trained end-to-end in a supervised manner to classify a patient’s respiratory rate/pattern given the input Wi-Fi CSI data stream. The tuple (Re(**h**_*n*_), Imag(**h**_*n*_), *R*_*n*_) denote the *n*-th training sample in the training data set. In this paper, we consider the ground truth data to be data generated or output by the calibrated breathing manikin (described in Section V). This calibrated output includes respiratory motion, tidal volume, and respiratory rate. The cost function of interest is the cross-entropy loss between the ground truth value and the corresponding estimated respiratory motion value using the *BreatheSmart* network. Based on the loss value, the weight matrices are adjusted.

After *BreatheSmart* network is trained, the test Wi-Fi CSI data streams in 𝒩_test_, will be fed into the *BreatheSmart* network and utilized to directly predict/estimate the respiratory motion-related values. To be specific, each Wi-Fi CSI data stream **h**_*n*_, *n* ∈ 𝒩_test_, will be passed through the trained network that generates an output respiratory motion-rated value. This output will then be compared with the ground truth label to calculate the accuracy of the proposed algorithm.

### C. PERFORMANCE EVALUATION METRICS

The performance of the *BreatheSmart* algorithm is evaluated by obtaining a confusion matrix. The confusion matrix **C** for the respiratory motion classification problem is an *R* × *R* matrix that delineates the performance of the *BreatheSmart* classifier on a test set of CSI data streams while the target respiratory motion-related values are given. Based on the confusion matrix, the detected respiratory motion-related values are compared to the ground truth data. The rows (resp. columns) of the confusion matrix correspond to the predicted (resp. true) respiratory motion-related values, and diagonal (resp. off-diagonal) elements of the matrix **C** are correlated with Wi-Fi CSI data streams (observations) that are correctly (resp. incorrectly) classified. The confusion matrix provides information on true/false positives (TP/FP) and true/false negatives (TN/FN). For each class, the true positive (resp. true negative) equals the correctly identified (resp. rejected) respiratory motion-related value prediction, while the false positive (resp. false negative) equals the incorrectly identified (resp. rejected) respiratory motion-related value prediction. For the *r*-th respiratory motion-related value (the *r*-th class, i.e., *R*_*r*_), where *r* ∈ ℛ = {1,·⋯, *R*}, these values can be calculated as:

(6) $${\text{TP}}_{r}=C_{rr},\;{\text{FP}}_{r}=\sum_{\begin{array}{c} i=1\\ i\neq r \end{array}}^{R}C_{ri},\;{\text{FN}}_{r}=\sum_{\begin{array}{c} i=1\\ i\neq r \end{array}}^{R}C_{ir},\;{\text{TN}}_{r}=\sum_{i=1}^{R}\sum_{j=1}^{R}C_{ij}-\left({\text{TP}}_{r}+{\text{FP}}_{r}+{\text{FN}}_{r}\right).$$

These values are then utilized in calculating the classifier performance metrics such as accuracy, precision, recall, specificity, and F1-score [46]. The model’s overall accuracy indicates how often respiratory motion-related values, i.e., RRs or respiratory patterns, corresponding to Wi-Fi CSI data streams are correctly identified out of all possible classes. This can be calculated as:

(7) $$\text{Accuracy}=\sum_{r=1}^{R}{\text{TP}}_{r}/\sum_{i=1}^{R}\left({\text{TP}}_{r}+{\text{TN}}_{r}+{\text{FP}}_{r}+{\text{FN}}_{r}\right)\;=\sum_{i=1}^{R}{\text{C}}_{ii}/\sum_{i=1}^{R}\sum_{j=1}^{R}{\text{C}}_{ij}.$$

We are also interested in calculating the precision and recall values for each respiratory pattern or rate class. While for a given respiratory motion-related value (class) *r*, precision tell us how many Wi-Fi CSI data streams were correctly predicted–out of the times label *r* was predicted, “precision × 100%” of the time the *BreatheSmart* classifier was correct–i.e.,

(8) $${\text{Precision }}_{r}={\text{C}}_{rr}/\sum_{i=1}^{R}{\text{C}}_{ri},$$

the recall value, also known as sensitivity, tells us that for all Wi-Fi CSI data streams that are classified as *r* class, how many of these were correctly captured, i.e., out of all the times the class value *r* should have been predicted for a given Wi-Fi CSI data stream only “recall × 100%” of the predicted labels were correctly predicted

(9) $${\text{Recall }}_{r}={\text{C}}_{rr}/\sum_{i=1}^{R}{\text{C}}_{ir}\text{. }$$

The specificity metric tells us what fraction of all Wi-Fi CSI data streams that do not belong to the *r* respiratory rate/pattern class are correctly predicted as non *r* class by the classifier and can be calculated as

(10) $${\text{Specificity }}_{r}=\sum_{\begin{array}{c} i=1\\ i\neq r \end{array}}^{R}\sum_{\begin{array}{c} j=1\\ j\neq r \end{array}}^{R}{\text{C}}_{ij}/\sum_{i=1}^{R}\sum_{\begin{array}{c} j=1\\ j\neq r \end{array}}^{R}{\text{C}}_{ij}\text{. }$$

To assess the quality of the proposed model, we also calculate the F1 score for each class. This metric can be calculated as follows:

(11) $$\text{F1 }{\text{score }}_{r}=2\times \frac{{\text{Precision }}_{r}\times {\text{Recall}}_{r}}{{\text{Precision}}_{r}+{\text{Recall}}_{r}}\text{. }$$

There are a few ways of calculating the classifier’s overall precision, recall, specificity, and F1 score. This paper uses the most common approach in which the arithmetic mean of the per-class precision/recall/specificity/F1 score is calculated. This approach is known as the macro-averaged precision/recall/specificity/F1 score or the macro-precision/recall/specificity/F1 score for short.

Though we can numerically calculate the accuracy of the prediction from our LSTM algorithm, this evaluation does not consider the clinical value of the information. For example, if a respiratory rate of 15 breaths per minute (BPM) is predicted from the algorithm, but the real value is 14 BPM, this will count as an incorrect classification in the confusion matrix and other metrics. Specifically, from the perspective of “classification”, identifying 15 BPM versus 14 BPM is flawed because there is only “right” and “wrong” classification. However, from a clinical perspective, the difference between 14 BPM and 15 BPM may not be significant, and even an “incorrect” value of 14 BPM may still be helpful– getting a *nearby* answer rather than a *far away* one.

From the perspective of classifying the respiratory pattern, we also examine the algorithm’s performance from a binary “normal” or “abnormal” classification. We do this in Section VI. This is done partly to relieve the algorithm from the strict obligation to choose a particular motion pattern and instead focus on the clinical value of knowing whether a patient has a normal breathing pattern or an abnormal one that may warrant further examination.

## V. EXPERIMENT SETUP

**FIGURE 5.**
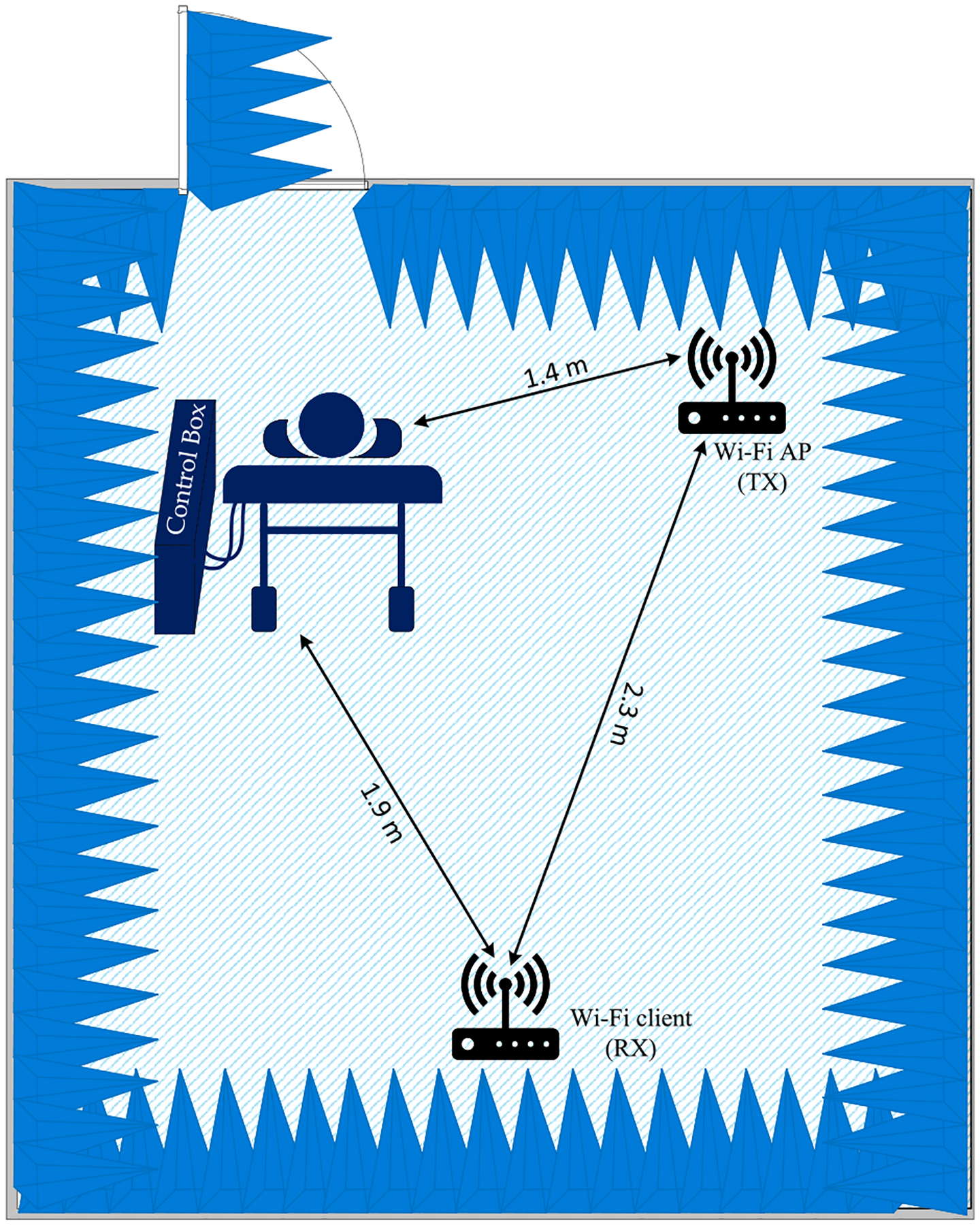
Physical layout of the measurement setup inside the anechoic chamber.

To characterize the performance of the algorithm proposed in Section IV, a series of physical experiments were designed and performed. These experiments were designed to utilize two Wi-Fi routers to send/receive the CSI signal, and a breathing manikin to serve as a surrogate for a human. In this work, no experiments were done with human or animal subjects. Since no trials involving humans were conducted, we had significant latitude in gathering data for the various scenarios. This ability provides access to a vast data set to train a stable, accurate, and reliable machine learning architecture that is hard to find in the literature due to the shortage of experimental data sets. The two Wi-Fi routers used were COTS devices with modified firmware loaded onto them. The modified firmware provided access to the raw CSI data and some parameters used to configure the CSI functionality (e.g., frame rate). In these experiments, Wi-Fi routers with an Atheros AR9344 chipset were configured with the CSI tool developed in [47]. These experiments aimed to examine the performance of the *BreatheSmart* algorithm under a variety of breathing patterns and rates, and path-loss conditions. All these experiments took place inside an anechoic chamber with interior dimensions of 3.0 by 2.3 by 2.1 meters (L, W, H; absorber tip to absorber tip). The hardware and manikin were arranged as shown in Figure 5. The dimensions shown in Figure 5 remained constant for all measurements.

**FIGURE 6.**
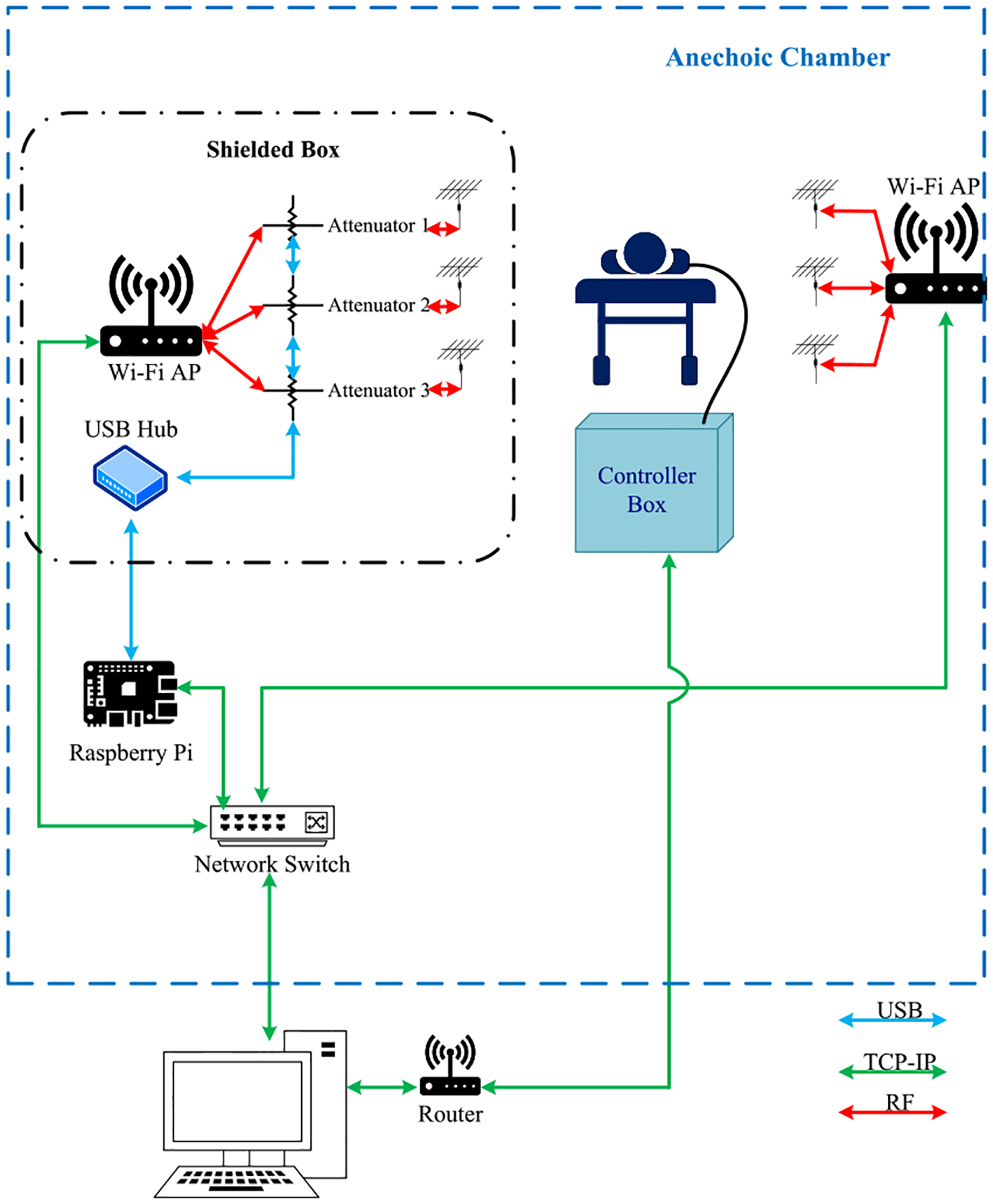
Block diagram of the measurement setup.

**FIGURE 7.**
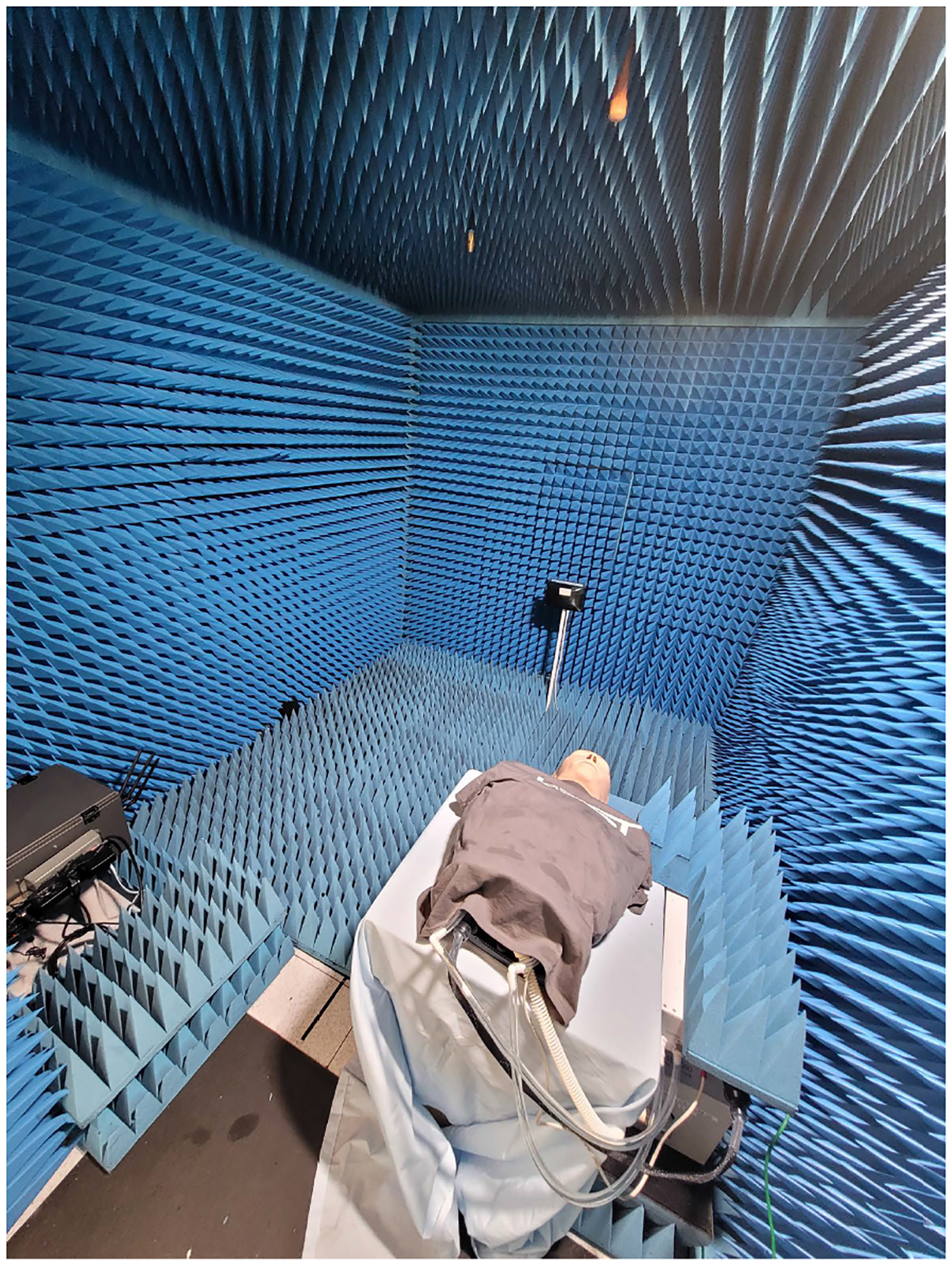
Photograph of the measurement setup. The breathing manikin can be seen on a table in the foreground with its control box under a piece of the absorber to the right. The small shielded enclosure for the transmitting Wi-Fi device can be seen on the left side (dark-colored box) with the three antennas connected to a bulkhead panel on the enclosure. The receiving Wi-Fi device is in the center of the image; placed on a pedestal in the back of the chamber.

A block diagram showing the connections of different pieces of hardware is shown in Figure 6, and a photograph of the measurement setup is shown in Figure 7. The Wi-Fi access point used as a transmitter (i.e., the one that sent the CSI signal) was placed inside a small shielded enclosure within the anechoic chamber. This was done to enable control of the power transmitted by the Wi-Fi device; something not easily possible through the firmware. Attached to each of the three antenna ports of the Wi-Fi transmit device was a variable attenuator that could be programmed via USB. The shielded enclosure ensured any leakage from the Wi-Fi transmitter was confined to the enclosure and did not reach the Wi-Fi client (receiver).

In this setup, the breathing manikin was connected to a controller unit that was part of an Ethernet network back to the control PC. A separate Ethernet network existed between a single board computer (i.e., Raspberry Pi), the Wi-Fi transmitter, and the Wi-Fi receiver. The single-board computer ran a Linux-based operating system and was connected via USB to the three variable attenuators on the transmitting Wi-Fi AP and was used to control the Wi-Fi devices (AP and client) during the test, manage automated data acquisition, and gather raw CSI data. It communicated with the device’s firmware to set the transmit parameters of the CSI signal and read the CSI data frames from the receiving device.

A control PC was used outside the anechoic chamber to remotely connect to the single board computer, configure it for automated measurements, and to off-load raw CSI data from the single board computer at the end of a measurement. This control PC was also used to process the raw CSI data as described in Section VI.

### A. BREATHING MANIKIN

In work presented here, we opted to use a calibrated breathing manikin, known commercially as the “RespiPro.” According to the manufacturer, the manikin features an anatomically correct airway and chest structure paired with high-fidelity spontaneously breathing lungs [48]. An Active Servo Lung (ASL) 5000 Breathing Simulator (model 3100150) [49] is connected to the manikin and is responsible for creating the airflow that simulates breathing. This manikin is typically used to train medical professionals on various patient procedures (e.g., chest tube insertion, intubation). With software provided by the manufacturer, the breathing pattern, rate, and tidal volume (TV) can be controlled. Tidal volume denotes the amount of air displaced or exchanged in each respiratory cycle, and it is a vital clinical parameter in setting the ventilator in patients [50]. The user can select from pre-defined respiratory patterns that are associated with a variety of medical conditions.

In this case, we have omitted the medical disease or disorder associated with each pattern and instead refer to them as patterns #1−#9, as shown in Table 1. The patterns in Table 1 were selected in an attempt to accentuate the differences in pattern and tidal volume range. By maximizing the difference in patterns and tidal volumes, we present our proposed algorithm with a larger variety of respiratory patterns to classify. This will make for a more thorough evaluation of the proposed technique. The respiratory motion of the breathing manikin is physically calibrated by the manufacturer at regular intervals (e.g., annually).

## VI. CLASSIFIER PERFORMANCE ASSESSMENT

In this section, we evaluate the performance of our proposed *BreatheSmart* algorithm in terms of identifying breathing patterns and rates. The proposed algorithm is implemented in MATLAB R2021b on one computer node with a 4-core central processing unit (CPU), with 40 GB of RAM, and one graphical processing unit (GPU) capable of approximately 863 GFLOPS with 2 GB of dedicated RAM.

### A. TRAINING AND TEST DATA SETS

As discussed in Section V, we first selected nine breathing patterns to classify respiratory pattern motions. Among these nine breathing patterns, one is associated with *normal* breathing^3^ while the rest —called *abnormal* breathing patterns in work presented here—are associated with scenarios in which the patient under test is breathing either more slowly or faster than *normal*, or there is an increase/decrease in the ventilatory response. Each configuration is one pattern tested at a single RR, as depicted in Table 1. As discussed in Section III, each pattern was collected for a Wi-Fi frame rate of 10 and run for 60 seconds while Wi-Fi CSI data streams were collected hereafter, otherwise specified. Specifically, for each pattern, we collect CSI data streams from 2 transmit antennas, 3 receive antenna, and 56 sub-carriers— 336 number of data streams per pattern and overall 3024 data streams in the data set. Since the ability to accurately detect and classify an abnormal breathing pattern can remarkably extend the usefulness of this technique, we grouped all abnormal breathing patterns– and their associated 2688 CSI data streams– and classified them as abnormal. To ensure hat the data set is not biased, we collect 2352 more CSI data streams associated with normal breathing. Therefore, our data set for classifying a normal vs. abnormal breathing pattern contains 5376 CSI data streams.

As for the RR classification, we consider the breathing rates associated with three breathing patterns: Eupnea (12–20 BPM), Bradypnea (3–11 BPM), and Tachypnea (21–30 BPM) [51]. We treat increments of BPM, i.e., from 3 to 30, as separate classes divided into integer increments^4^–overall 28 classes. The configuration for each class was run for 60 seconds, and the frame rate that the CSI data stream was being transmitted was set to 10 frame per second while data were collected, otherwise specified. The data set for classifying the RR consists of 9408 CSI data streams for 28 breathing rates.

To split each of the data sets mentioned above into the training and test data sets, we consider a 10-fold cross-validation and calculate the standard deviation of overall accuracy. Standard deviation is an essential factor in evaluating *k*-fold cross-validation since it summarizes the expected variance in the classifier’s performance. For each classification problem of interest, we first shuffle the corresponding data set– and their accompanying label– randomly. Shuffling data set ensures that the model is not overfitting to a specific pattern/rate and preserves a class balance between train and test sets. Specifically, the data sets–combined of 3024, 5376, and 9408 CSI data streams, which each runs for 60 second for classifying breathing patterns, abnormal breathing, and respiratory rate, respectively– are sorted by patterns’ number/BPM values; hence, the model would fit satisfactorily on the first sets of patterns/rates and overfits the rest. Subsequently, we split the data set into 10 groups for each data set. Afterward, each distinctive group is considered a holdout (test data set), while the remaining groups are regarded as a training data set. For each set of the training and test data sets, we fit the *BreatheSmart* learning model on the training set; that is, the entire training data set is used to optimize the weights of the proposed deep neural network. We choose the number of neurons (50), learning rate (0.01), and batch size (64) parameters by tuning the hyperparameters. In the testing stage, we evaluate the classifier’s performance metric on the test data set– we pass each Wi-Fi CSI data stream in the test data set through the trained *BreatheSmart* network and collect the predicted class at the output and compare the result with the target label to calculate the classifier’s performance metric. We also calculate the standard deviation of overall performance metrics. Using 10-fold cross-validation, each Wi-Fi CSI data stream is allowed to be used in the test set once and goes towards training the network 9 times.

The reason for the classification of “normal” vs. “abnormal” breathing patterns can be viewed as an alternative way of assessing the algorithm’s output. Because in the specific respiratory pattern classification problem, the pattern is classified as a specific pattern, an incorrect classification may be unnecessarily harsh. Even if the algorithm cannot identify the specific breathing pattern, there may still be some value if the algorithm can identify the pattern as something other than “normal”. The definition of “normal” and “abnormal” can be adjusted to fit a specific application.

In each classification problem, each Wi-Fi CSI data stream will be associated with one respiratory rate/pattern using the *BreatheSmart* algorithm, and the ground truth motions-related values programmed into the calibrated breathing manikin are used to measure the algorithm’s accuracy. The classification performance was assessed by obtaining a confusion matrix.

**FIGURE 8.**
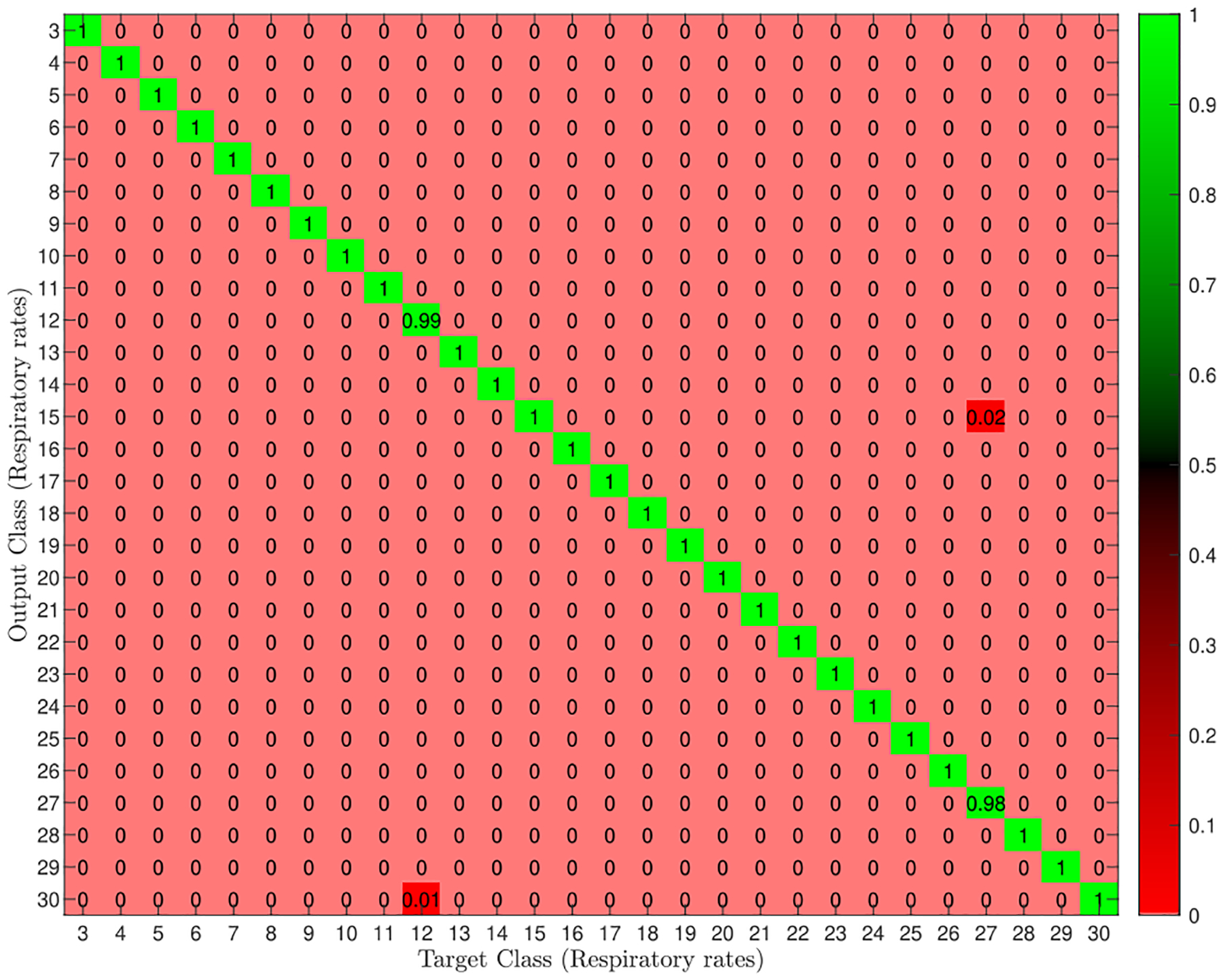
Breathing rate classification for Att = 0dB and T = 60 sec. Each class corresponds to a respiratory rate, ranging from 3 BPM to 30 BPM. The misclassified predictions happen at (30, 12) and (15,27).

The performance of the proposed classifier in classifying respiratory rates for the scenario in which a line-of-sight path between the manikin and the transceiver pair is available was assessed by computing the confusion matrix. Figure 8 shows the heat-map of the normalized confusion matrix. Each cell (*i*, *j*) represent the probability of class *j* label were predicted as class *i*. The lighter the red color, the smaller the misclassification rate. The results indicate that when a line-of-sight path is utilized between the transmitter and receiver, 99.97% of the Wi-Fi CSI data streams are correctly assigned to the corresponding respiratory rates, while 0.03% of respiratory rate predictions based on CSI are incorrect.

**FIGURE 9.**
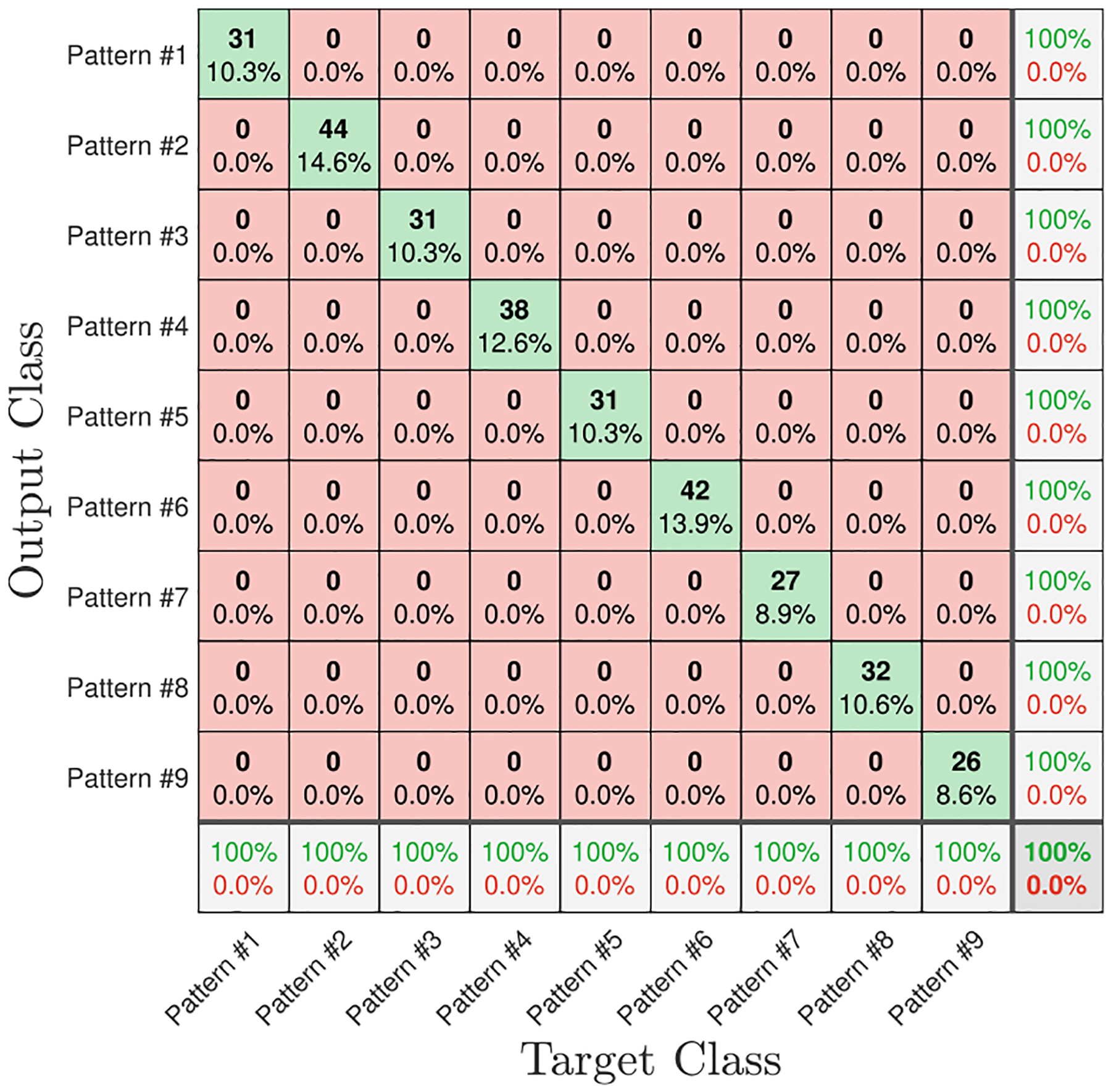
Breathing pattern classification for Att = 0dB and T = 60 sec.

Figure 9 demonstrates the confusion matrix —for 1-fold of cross-validation— of the pattern classification problem for a perfect scenario in which a line-of-sight path between the manikin and the monitor is available, and each breathing pattern was held for 60 seconds while data were collected. As shown in Figure 9, the measurements belonging to all breathing patterns are classified correctly when zero additional path loss is added to the measurement circuit. Diagonal elements of the confusion matrix show the number and percentage of correct classifications by the *BreatheSmart* algorithm. For instance, 38 CSI streams are correctly classified as Pattern #4, corresponding to 12.6% of all 302 CSI streams. The overall number of data streams, i.e., 302, is equal to the data streams in the test data set, which equals 10% of the whole data set.

These results are promising but reflect measurements taken in an ideal environment: low RF path-loss (high SNR) and a high CSI frame rate. Later in this section, we will analyze how the algorithm performs as these two parameters are varied. Note that other parameters can impact the estimation, such as the patient’s physical position, the physical location of the patient relative to the Wi-Fi client and access point, dynamic movement in the environment, and the RF reflectivity of the environment. While these additional factors are outside the scope of this work, the methods outlined here can be used to quantify their impacts in future work.

### B. CLASSIFICATION PROBLEMS OF INTEREST

We consider the following classifications problems:

- Respiratory rate classification
- Respiratory motion classification
  - Specific respiratory pattern classification
  - Normal vs. abnormal breathing pattern classification

### C. ATTENUATION EFFECT ON THE CLASSIFICATION ACCURACY

To evaluate the impact of RF propagation path-loss on the ability of the algorithm to classify respiratory pattern motion and rate correctly, we artificially added attenuation between the Wi-Fi client and access point in the anechoic chamber. This effect could also have been achieved by increasing the physical distance between the Wi-Fi client and the access point.

As an approximate translation between path-loss and physical distance, we can use the formula for free-space path-loss (FSPL). The FSPL is considered here to predict the CSI signal strength in the setup mentioned above. The FSPL can be expressed as follows

(12) $$\text{FSPL}={\left(4\pi df/c\right)}^{2}$$

where *d*, *f*, and *c* denote distance from the transmitter to receiver in meters, signal frequency in hertz, and the speed of lights in meters per second, respectively. Since we assume identical omnidirectional antennas in each instance, antenna gains at the transmitter and receiver do not have any reflection on computing relative changes in distance. The relationship between the distance, attenuation, and path-loss is specifically mentioned in Table 2. The values in Table 2 are only an approximation. Doors, windows, walls, and other reflective or lossy objects will influence the path-loss *without* increasing the distance between the Wi-Fi devices and the patient.

**FIGURE 10.**
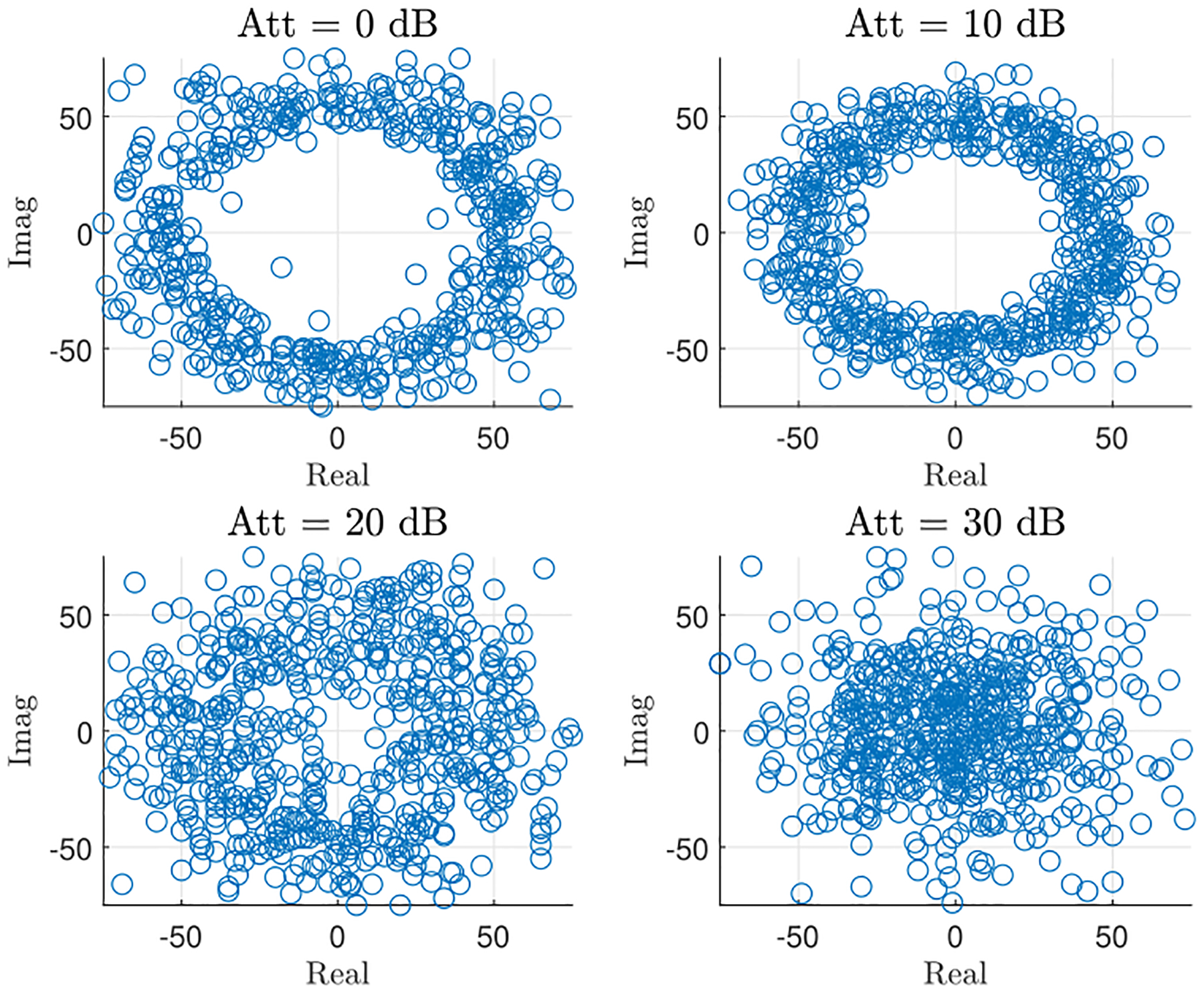
Effect of attenuation on a complex CSI real and imaginary parts of a normal breathing with RR = 15 BPM.

**FIGURE 11.**
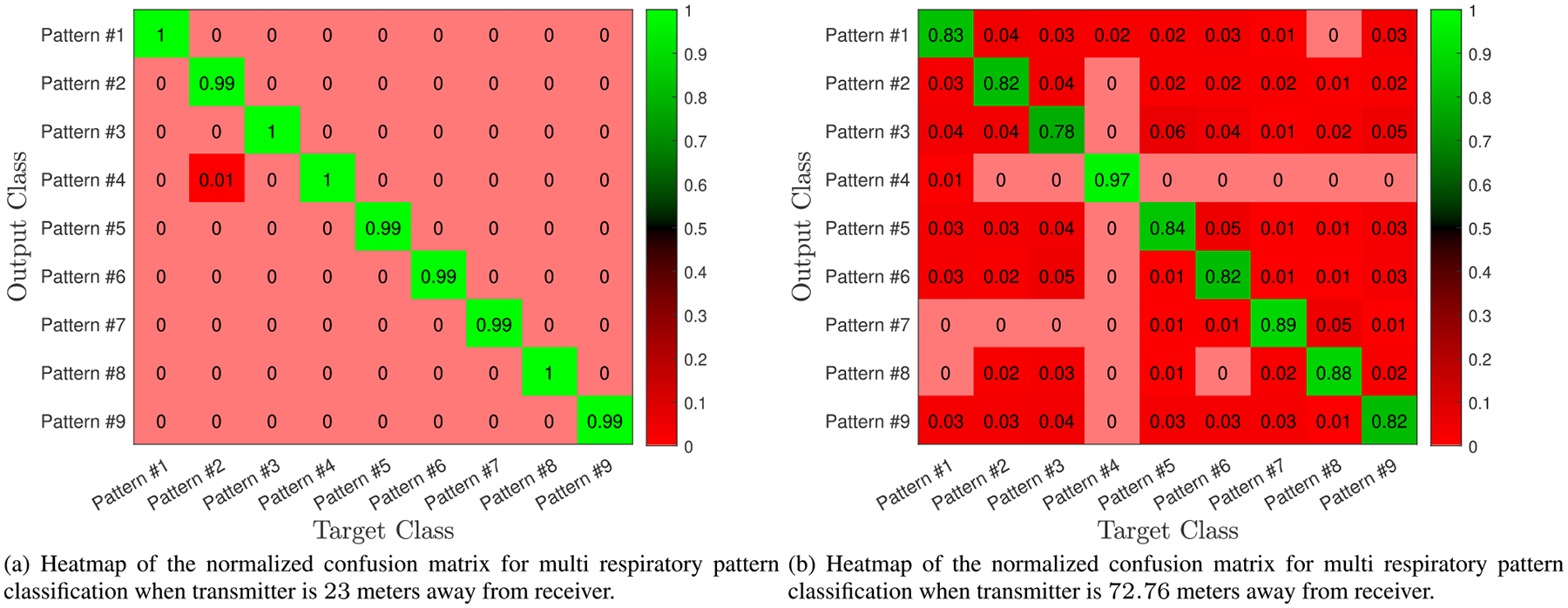
Heatmap confusion matrices. The rows and columns of the confusion matrix correspond to the predicted and true classes, respectively. Column-normalized elements display the percentages of correctly and incorrectly observation for each target class.

Figure 10 demonstrates how attenuation impacts the CSI data streams and the respiratory information in the signal. By changing the attenuators, we can then calculate the distance effect on the classifier’s performance metrics, as listed in Table. 3. These results show that the classification accuracy degrades as the CSI signal-to-noise ratio (SNR) decreases. Specifically, the accuracy drops from 100.00% to 85.02% when the distance corresponding to the investigated attenuation increases from 2.3 to 72.76 meters. These results highlight that the difficulty in classifying the signal increases as the SNR of the input data decreases. Eventually, the SNR will become so poor that the accuracy of the classifying algorithm will fall below a useful level. Figure 11 (a) and (b) show the heat-map of the normalized confusion matrix (each element of this matrix is rounded to the nearest integer) for the scenarios in which the transmitter is at an equivalent distance of 23 and 72.76 meters away from the receiver, respectively. For attenuation loss of 20 dB, all respiratory patterns are very well classified with above 99% correct classification. As we artificially increase the distance between the transmitter and receiver, things get more complicated as at high attenuation most breathing patterns look alike. Therefore, the ability of the algorithm to accurately classify each respiratory pattern motion drops to above 78% for all classes. Nevertheless, the classification accuracies are not the same for all patterns. For instance, Pattern #4 is well classified, with 97% correct classification while Pattern #3 is the most affected pattern by high attenuation loss.

**FIGURE 12.**
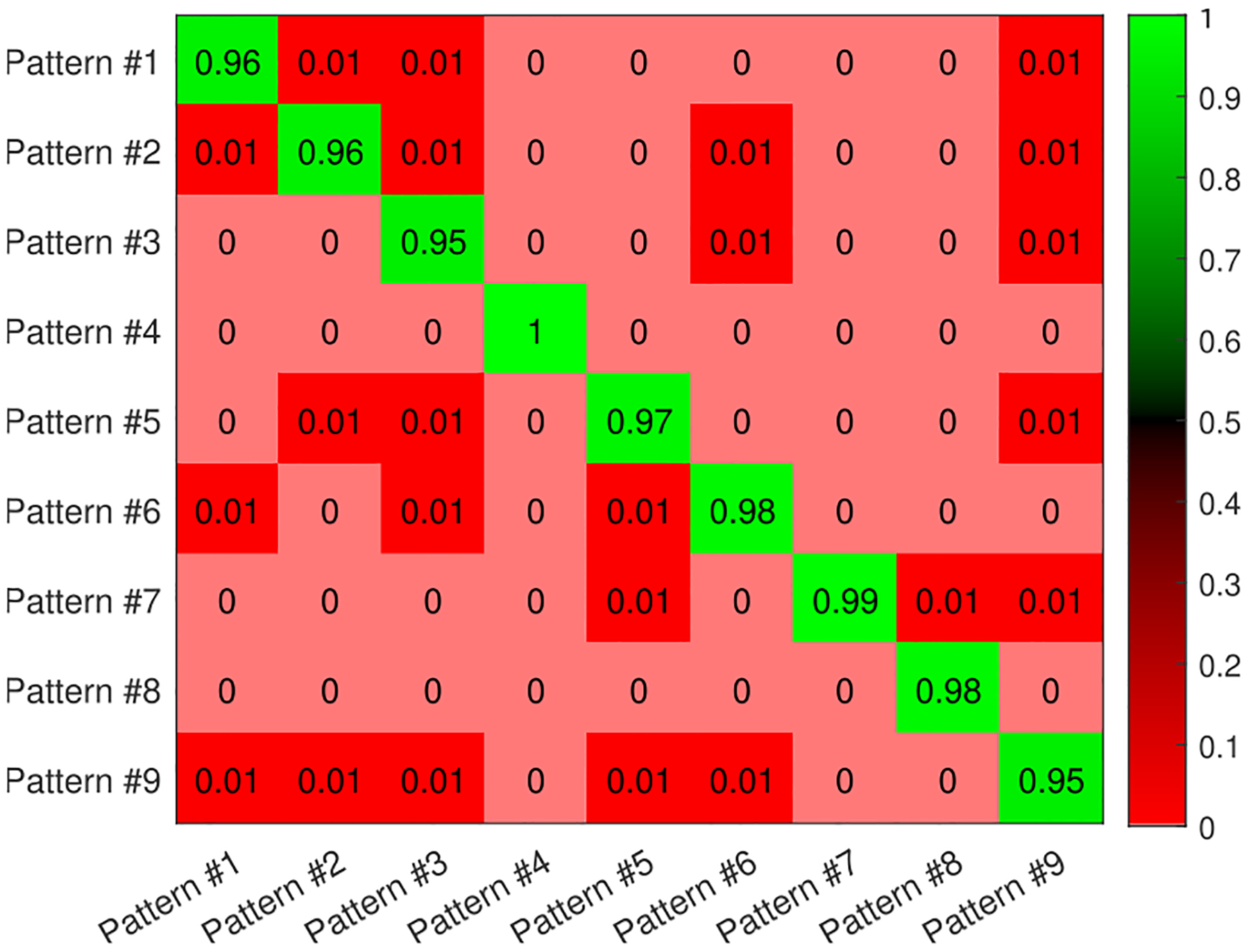
Heatmap of the normalized confusion matrix –with more data compared to Fig. 11 (b)– for multi respiratory pattern classification when the transmitter is 72.76 meters away from the receiver.

Increasing the amount of training data might improve the performance of the classifier. To evaluate the impact of data volume on accuracy, we acquired more data (double data set). We observed that overall pattern classification accuracy went up from 85.02% to 97.09% with (96.39%, 97.79%) of the standard deviation of the overall classifier model accuracy using the 10 folds for cross-validation. Moreover, the ability of the algorithm to accurately classify each respiratory motion goes up from about 78% to about 95% for all classes, as demonstrated in Figure 12.

As mentioned previously, when the pattern is classified as a specific pattern– as we did above, an incorrect classification may be unnecessarily harsh. Even if the algorithm cannot identify the specific breathing pattern, there may still be some value if the algorithm can identify the pattern as abnormal. For that reason, we consider the possibility of accurately classifying the breathing pattern either as normal or abnormal since the ability to accurately detect and classify abnormal breathing patterns may potentially extend the value of this technique. The results are summarized in Table 4 and show that in classifying the breathing pattern as normal vs. abnormal, the classifier accuracy only slightly drops–without increasing the amount of data– even when the equivalent distance between the manikin and the access point increases considerably.

Table 5 shows that the breathing rate classification results follow the same trend as pattern classification results concerning the manikin’s location and the transmitter-receiver distance. The classification accuracy degrades as the attenuation (i.e., path-loss) increases. However, compared to the respiratory pattern classification, there is a significant drop in accuracy as the equivalent distance between transmitter and receiver increases to 72.76 meters. This happens since the respiratory rate classification problem includes more classes (i.e., 28) than that of the respiratory pattern classification problem (i.e., 9). We also observed that, for a 30 dB attenuation, the testing accuracy converges to 76.81%– significantly lower than the training accuracy of 89.59%. This being the case, we acquired more data to see how the performance of our algorithm could be improved with more data. Results show that the testing accuracy increases notably for 30 dB of attenuation loss from 76.81% to 87.90%, with (87.23%, 88.56%) of the standard deviation of the overall classifier model accuracy using the 10 folds for cross-validation, when we double the volume of our data set.

To estimate how relevant these results are to real-world scenarios and to test the robustness of the proposed learned model, we consider a scenario in which the Wi-Fi CSI data streams included within the data set are affected separately with different manikin locations and the transmitter-receiver distance– different attenuations ranging from 0 dB to 30 dB– for all breathing patterns. In this scenario, the standard deviation of the overall classifier model accuracy for the proposed model using 10 folds for cross-validation equals (92.13%, 93.72%). The 10 fold cross-validation standard deviation of model accuracy goes up to (95.61%, 96.74%) when classifying respiratory motion as normal vs. abnormal. The same approach was chosen to collect the data set for calculating the robustness of the proposed algorithm with respect to the breathing rate classification corresponding to Wi-Fi CSI data streams that are attenuated separately. The standard deviation of the overall classifier model accuracy for the proposed model using 10 folds for cross-validation for this scenario equals (88.25%, 89.11%)– performance will increase with more data. We are also interested in investigating our proposed system’s ability to distinguish between three broad classes of respiratory rates, i.e., respiratory rates below 11 BPM, respiratory rates between 12 and 20 BPM, and respiratory rates above 21 BPM. Results show that the overall system classification accuracy standard deviation using 10 folds for cross-validation for this scenario equals (87.59%, 89.13%).

### D. EFFECT OF ACQUISITION LENGTH ON CLASSIFICATION ACCURACY

An additional factor to consider is how much CSI data (in time) is needed to correctly classify the respiratory pattern and rate. The acquisition length– the time of the CSI data stream being processed– can have two different impacts. Hence, the CSI signal length can be interpreted as an essential parameter in studying the *sensitivity* of the algorithm. The required CSI signal length for an accurate respiratory motion/rate classification can vary depending on the signal processing method. The performance of the *BreatheSmart* algorithm in classifying the respiratory rate and respiratory pattern for different CSI signal duration are demonstrated in Table 6 and 7, respectively, to understand their effects on proposed method. For each time duration– signal length– the accuracy of the respiratory pattern/rate classification algorithm is demonstrated. As shown in Table 6, by reducing the CSI signal length, the accuracy of motion classification is degraded since the frequency resolution in our pre-processing data method, i.e., FFT, is directly proportional to the CSI signal length. We found that the pattern classification accuracy of our algorithm slightly depends on the length of CSI signals. Table 7 depicts the influence of CSI signal length on the system performance of respiratory rate classification. The network accuracy follows the same trend as respiratory motion classification in classifying the respiratory rates.

### E. FRAME RATE EFFECT ON THE CLASSIFICATION ACCURACY

A critical aspect in estimating the respiratory motion/rate based on the Wi-Fi CSI data stream is the frequency of Wi-Fi frame transmissions carrying CSI data. Reducing the frame rate decreases the number of Wi-Fi samples, which also reduces the computational cost of signal processing and data acquisition time. Therefore, in this subsection, we investigate the effect of frame rate on the accuracy of the proposed processing algorithm in respiratory pattern and rate classifications. To do so, we gradually decrease the frame rate to see how the network accuracy is affected.^5^ Table 8 depicts the influence of Wi-Fi frame rate on the network performance of respiratory pattern classification. Notably, the classifier accuracy does not drop significantly as the Wi-Fi frame rate decreased from 10 frame per second to 3. Although we were unable to observe further degradation because of the system firmware limitations on how often we can transmit Wi-Fi frames, the classification accuracy is expected to decrease with further reductions of the Wi-Fi frame rate. The rationale behind this is that the frequency of all breathing patterns is less than the frame rate of 1 Hz, as it can easily be determined by Table 1, and to see the performance degradation, we need to consider the frame rate below 1 Hz, i.e., by dropping the frame rate down to 1/RR the performance should fall off significantly. However, the frame rate that the Wi-Fi CSI data was being transmitted cannot be set to a non-integer value– a value less than one frame per second. We discuss more this challenge in the limitations subsection.

### F. SIGNAL PROCESSING AND HARDWARE LIMITATIONS

In this Section, we discuss the observed or investigated limitations as part of our experiments and analyses. It may be possible to overcome some or all of these limitations, but doing so is outside our scope of showing how they can be assessed.

**FIGURE 13.**
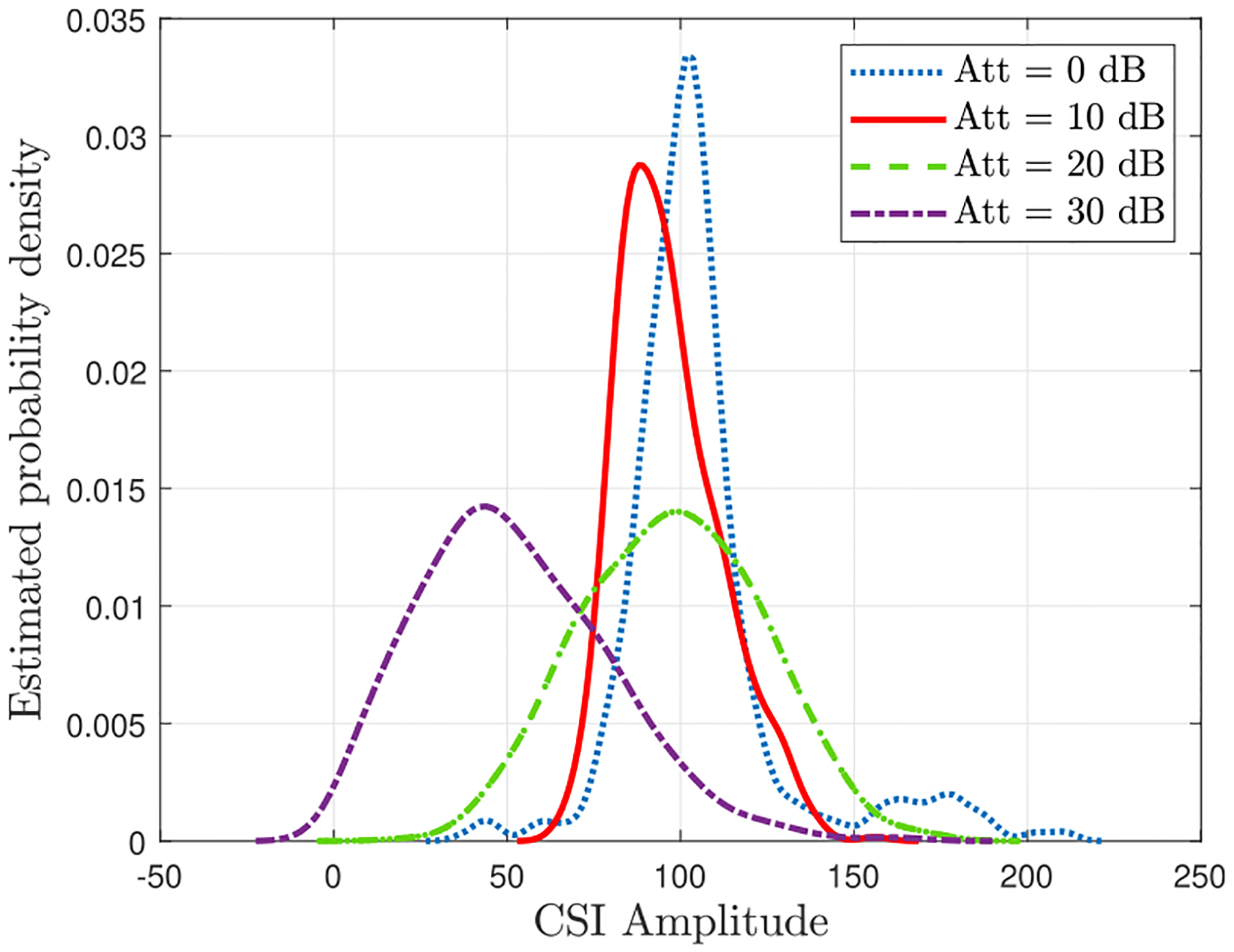
PDF estimation of attenuated Wi-Fi CSI data streams based on a normal Kernel function. Each line represents the PDF of Wi-Fi CSI amplitude values at a given attenuation value.

- Breathing manikin: In work presented here, a breathing manikin was used. This provides a bench method to develop and perform an early evaluation of monitoring methods (e.g., Wi-Fi configurations and algorithm designs) and investigates how different factors might affect the performance without needing human subjects. While human subjects could be used for this, the bench configuration provides a more rapid and straightforward approach for early design work. We can also set known conditions with the manikin compared to human subjects, where we would need to have subjects wear a reference sensor (e.g., clinically annotated capnography) to gauge the accuracy of the processing algorithm. In addition, care would need to be taken to ensure that the different human subjects breathe at various rates to ensure the performance of the processing algorithm is thoroughly characterized.
  One drawback to using the breathing manikin instead of a human subject is that the dielectric properties - primarily the RF reflectivity - of the manikin may or may not be similar to that of a human. The manikin appears to be made with materials (e.g., plastic, silicone) that are not reflective from an RF perspective, but slight differences between the reflectivity of a human subject and the breathing manikin may exist. Here, we did not investigate differences in reflectivity between human subjects and the breathing manikin to understand the potential impact on the CSI data and algorithms.
- Limitations of the signal processing algorithm: As discussed above, the performance accuracy of the proposed method in classifying the respiratory rates and patterns drops when we increase the equivalent distance between the transmitter and receiver. This was evident when the free space path-loss was increased to 30 dB, corresponding to 72.76 m. However, Results show that using more data helps overcome this limitation. This limitation is common in learning-based algorithms.
- RF dynamic range: One limitation in increasing the equivalent distance or path-loss between the transmitter and receiver is that as we move the transmitter more than 102.78 m away from the receiver, the path-loss can become too high to create a link. Therefore, when attenuation between the Wi-Fi client and access point in the anechoic chamber is set to a higher value– above 33 dB– the link cannot be established, and the receiver does not reliably receive CSI data. Establishing this limit is important because it gives an idea of how much attenuation the system can tolerate before its use becomes impractical.
- Number of data streams: The inherent variability of real data was observed through our extensive measurement. For example, we noticed that the 3rd transmit port only worked intermittently in our system. Therefore, we took the data stream associated with the 3rd transmit antenna off and only processed the CSI data streams acquired from the first two transmit antennas. More advanced algorithms could include the ability to select which data stream(s) to use in its processing.
- Detecting Apena: One respiratory pattern not represented in Table 1 features apnea. The breathing manikin can produce a pattern with apnea, but we had difficulty seeing the absence of breathing in the CSI signal. We found that even when there was not any breathing - and thus no movement inside the anechoic chamber - we were still seeing what appeared to be an affected CSI signal by breathing. To investigate, we generated a plot of probability density functions (PDF)s of CSI data obtained from the manikin breathing in a normal pattern but with increasing attenuation levels (Figure 13). We hypothesize that, at 0 dB and 10 dB of attenuation, we are saturating the receiving Wi-Fi client with a strong signal. Once we get out of the saturation region (20 dB and 30 dB), we see that the peaks of the PDFs of Wi-Fi CSI amplitude are closely matched. This shows that even though attenuation between the Wi-Fi client and access point increases, the reported amplitude of the Wi-Fi CSI signal does not. We suspect that the Wi-Fi receiver is doing equalization or amplifying the received CSI signal, known as automatic gain control (AGC), making detecting the absence of movement difficult. In essence, when the manikin is not moving, and we would expect a “quieter” Wi-Fi CSI response, the receiving Wi-Fi client amplifies the signal to keep the amplitude up. This issue could be confirmed and overcome if one had access to the AGC registers on the Wi-Fi client chipsets; however, the low-level control is not accessible to us.
- Enumerating patterns and rates: One challenge to deploying this technique in the real world is the training data set to train the neural network. The algorithm needs to be trained on each respiratory rate, pattern, and a combination thereof that may be encountered to be most effective.
  One approach to enumerating all the rates and patterns would be to dissect the technique further to understand which parameter in human breathing causes the most influence on the CSI data. In short, the CSI technique detects physical deflection or movement of the human torso. However, in this work, the *cause* of that deflection - be tidal volume, muscle pressure, or other factors - is not separated. They are treated as a single, confounded cause. Performing separate experiments where a single respiratory pattern is selected and the individual factors are manipulated could provide insight into how sensitive the Wi-Fi technique is to specific aspects of respiratory motion. This knowledge could then be used to develop a more complete training data set for the neural network.

## VII. CONCLUSION AND FUTURE WORK

We developed and presented the *BreatheSmart* algorithm, a robust respiratory rate/pattern classification technique that leverages the ubiquity of existing Wi-Fi infrastructure and deep learning techniques to detect human respiration motions based on Wi-Fi CSI data streams. Alongside this algorithm, we presented a thorough characterization of the system performance. The method used for the characterization can be used to assess the performance of similar CSI-based detection techniques. We studied the effect of pattern type, attenuation-loss, acquisition length, and frame rate on human respiratory motion and rate classification accuracy. Extensive laboratory experiments and data acquisition utilizing an example of respiratory motion monitoring based on Wi-Fi CSI demonstrated the capability of the proposed system in detecting and classifying this vital sign. Notably, the introduced deep learning algorithm could be applied to many types of communication systems, but we utilize Wi-Fi as an example.

Future work can explore several avenues. One is to incorporate the effects of additional parameters such as the patient’s physical position, physical location of the patient relative to the Wi-Fi client and access point, dynamic movement in the environment, and the RF reflectivity of the environment. The problem of multi-user respiratory motion detection in which multiple people are in the same room can also be explored. One way to explore the impact of the environment would be to perform measurements inside an empty or loaded reverberation chamber, which provides a highly reflective environment in contrast to the reflection-less anechoic chamber used in this work. The reverberation chamber simulates more multi-path reflections, and if continuously stirred, simulates an environment with dynamic fading.

Another avenue for future research is to add a wireless coexistence test to help quantify the impact and reliability of this technique operating in a crowded spectrum environment. Coexistence scenarios in which multiple wireless protocols share the same spectrum band are common. These coexisting signals can interfere with each other and cause one or more devices to fail to maintain their functionality. CSI signals are vulnerable to complex environments and coexistence makes this vulnerability even more severe since Wi-Fi CSI can be easily contaminated by interference from other wireless standard protocols working on the same spectrum. We are interested in testing the reliability of our algorithm by doing a coexistence test based on measurement methods found in [52].

Finally, one could conceivably apply the *BreatheSmart* algorithm to other wireless protocols with a channel-sounding signal. For example, the sounding reference signal used in 4th Generation Long-Term Evolution (4G LTE) or 5th generation New Radio (5G NR) cellular technologies could be used to detect respiratory motion. The challenge with these protocols is that they’re typically used to sound channels over a larger distance than is commonly found between a Wi-Fi AP and client device. However, this technique may be useful for small cells.

As an alternative to cellular-type signals, the next generation of Wi-Fi technology (e.g., IEEE 802.11 ax and beyond) incorporates beamforming and will likely utilize CSI signals on a *per beam* basis. This adds an additional dimension to this type of detection and may aid in isolating the respiratory motion of interest when there are multiple people in a room or in a dynamically changing environment.

The current work of the IEEE 802.11 standards group suggests that they are aware of the ability to use CSI signals for motion detection. The IEEE 802.11 working group approved a new task group called IEEE 802.11bf Task Group (TGbf). This task group works on defining modifications to the state-of-the-art IEEE 802.11 standards at both the physical and medium access layers to enhance Wi-Fi sensing applications operation in the license-exempt frequency band between 1 GHz and 7.125 GHz and above 45 GHz [53]. Though the work of that task group is in parallel with the work presented here, part of the goal of this work is that it can be implemented on existing infrastructure. It may still be several years before Wi-Fi access points and devices that support IEEE 802.11bf become ubiquitous.

## Figures and Tables

**FIGURE 14.**
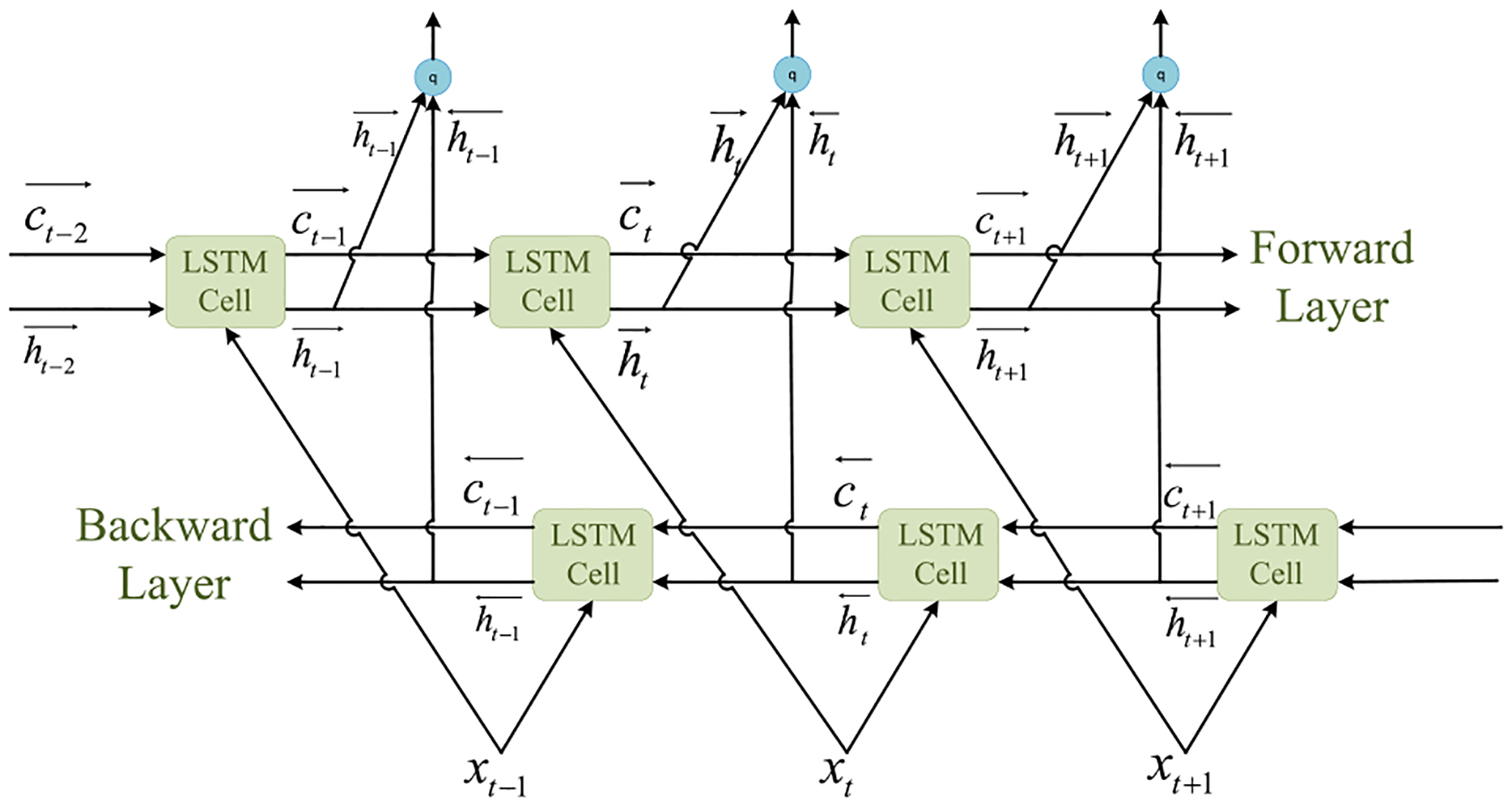
Bidirectional LSTM architecture.

**FIGURE 15.**
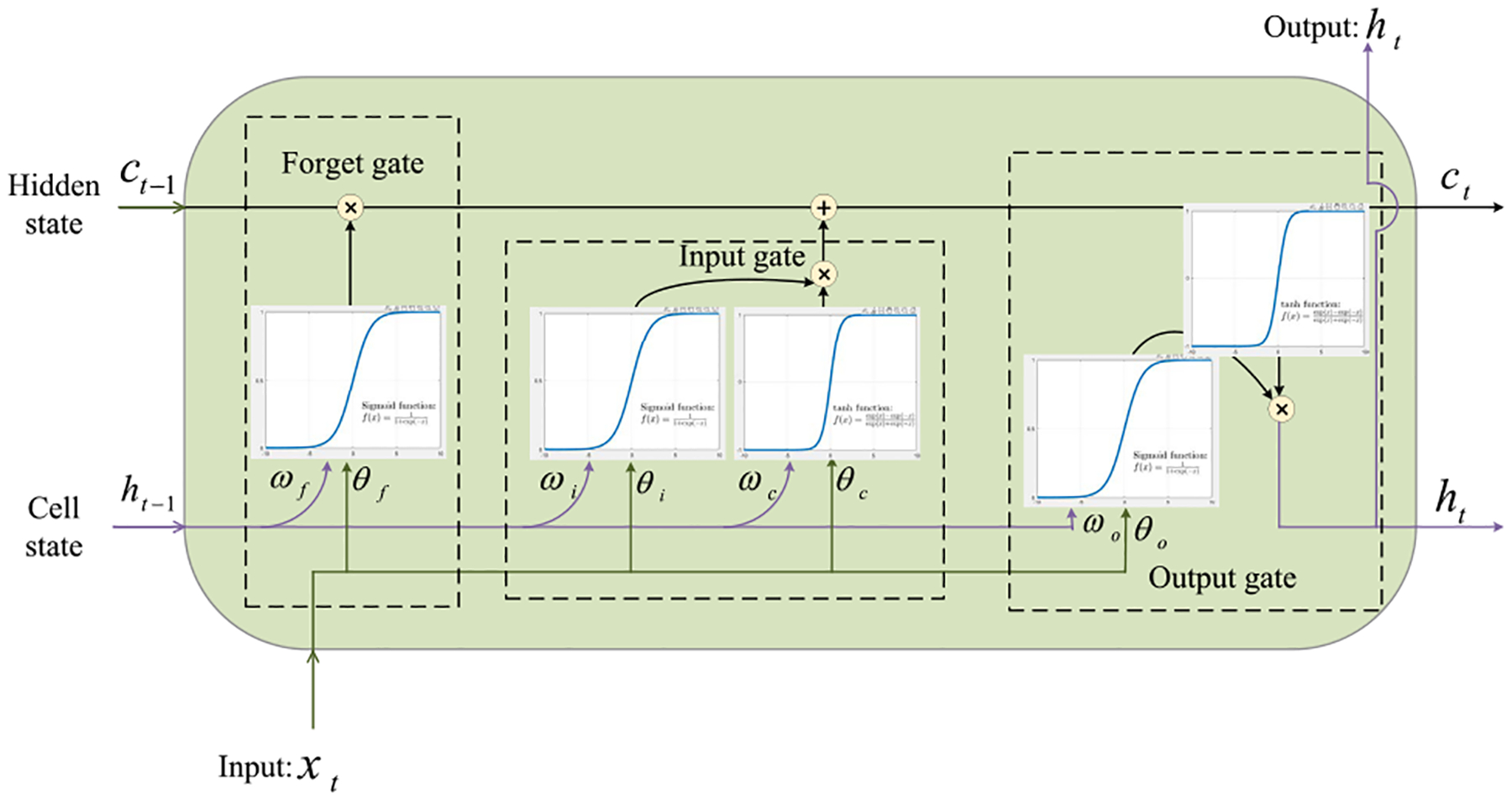
LSTM cell.

**TABLE 1.** Breathing patterns.

Pattern	BPM	Max Inspiratory/Expiratory TV (mL)	TV vs. time
#1	25	319	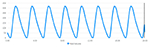
#2	6	705	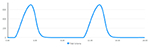
#3	18	488	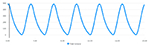
#4	18	171	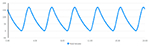
#5	22	651	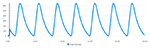
#6	15	587	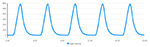
#7	28	442	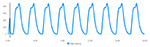
#8	19	513	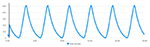
#9	25	463	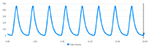

**TABLE 2.** A rough approximation of relative changes in distance. The value for 33 dB of attenuation is noted because this was the maximum amount of attenuation that could be applied and still have the Wi-Fi link operate.

Attenuation (dB)	Distance (m)	Path-loss (dB)
0	2.30	54
10	7.28	64
20	23.00	74
30	72.76	84
33	102.78	87

**TABLE 3.** Attenuation effect on the respiratory pattern classification accuracy for T = 60 sec. Each entry shows a point estimate with a 95% confidence interval on accuracy.

	Att = 0 dB	Att = 10 dB	Att = 20 dB	Att = 30 dB
Accuracy	100.00% (100%, 100%)	99.97% (99.89%, 100%)	99.54% (99.26%, 99.81%)	85.02% (83.66%, 86.38%)
Precision	100.00%	99.97%	99.58%	85.07%
Recall	100.00%	99.97%	99.56%	85.26%
Specificity	100.00%	99.99%	99.94%	98.13%
F1 score	100.00%	99.97%	99.57%	84.94%

**TABLE 4.** Attenuation effect on the normal vs. abnormal respiratory pattern classification accuracy for T = 60 sec. Point estimate and a 95% confidence interval on accuracy.

	Att = 0 dB	Att = 10 dB	Att = 20 dB	Att = 30 dB
Accuracy	99.94% (99.82%, 100%)	99.89% (99.76%, 100.00%)	98.77% (98.22%, 99.32%)	94.38% (93.05%,95.72%)
Precision	100%	99.78%	98.03%	95.76%
Recall	99.89%	100.00%	99.51%	93.19%
Specificity	100%	99.78%	98.06%	95.64%
F1 score	99.94%	99.89%	98.76%	94.46%

**TABLE 5.** Attenuation effect on the respiratory rate classification accuracy for t = 60 sec. Point estimate and a 95% confidence interval on accuracy.

	Att = 0 dB	Att = 10 dB	Att = 20 dB	Att = 30 dB
Accuracy	99.97% (99.92%, 100.00%)	99.88% (99.67%, 100.00%)	99.28% (99.09%, 99.47%)	76.81% (75.99%, 72.62%)
Precision	99.97%	99.88%	99.28%	76.97%
Recall	99.97%	99.88%	99.28%	76.81%
Specificity	100.00%	100.00%	99.97%	99.14%
F1 score	99.97%	99.88%	99.28%	76.82%

**TABLE 6.** CSI stream’s length effect on the respiratory pattern classification’s accuracy for attenuation loss = 20 dB.

	T = 30 sec	T = 15 sec	T= 10 sec
Accuracy	99.10%	98.13%	97.43%
F1 score	99.10%	98.13%	97.43%

**TABLE 7.** CSI stream’s length effect on the respiratory rate classification’s accuracy for attenuation loss = 20 dB.

	T = 30 sec	T = 15 sec	T = 10 sec
Accuracy	97.98%	96.10%	94.50%
F1 score	97.98%	96.10%	94.51%

**TABLE 8.** Wi-Fi frame rate (Frame/Sec) effect on the respiratory pattern classification’s accuracy for attenuation loss of 0 dB.

Accuracy\Frame rate	10	8	6	3
Accuracy	100%	100%	100%	99.83%

## Footnotes

1 The Fresnel zone is one of several confocal prolate ellipsoidal space regions between and around a transceiver. Due to obstructions or deflecting objects between the transmitter and receiver, a transmitted signal may take slightly different paths, reaching the receiver at distinct times with different phases. The calculated Fresnel zone size at any given distance from the transceiver determines whether these objects will cause notable interference.

2 Note that the complex CSI values are handled as a single value consisting of real and imaginary components. This is opposed to performing filtering operating separately on the magnitude and phase components.

3 The normal breathing pattern that we consider here involves a small inhalation followed by an exhalation that is succeeded by an automatic pause for about 1 to 2 seconds. It takes about 12 to 20 breaths per minute, and the tidal volume contains approximately 500 – 600 mL of air.

4 Note that we chose the classifier to report the results as discrete classes as opposed to numerical values.

5 Frame rates of less than 1 were not possible due to a firmware limitation. Attempts to simulate a frame rate of less than one by decimating the data produced unreliable results.

## Appendix

### APPENDIX A NETWORK ARCHITECTURE

As described in Section IV-C, our proposed algorithm uses an LSTM network. Its core components are a sequence input layer and a bidirectional LSTM (BiLSTM) layer. While the sequence input layer feeds time-series Wi-Fi CSI data stream **h**_*n*_ to the network, the BiLSTM layer learns bidirectional long-term dependencies between time steps of **h**_*n*_ over time. A BiLSTM layer consists of two LSTM layers– a forward LSTM layer and a backward LSTM layer– each made up of *N* LSTM hidden units, also known as LSTM cells, and processes data in both forward and backward directions as illustrated in Figure 14. By picking out the number of LSTM cells in each LSTM layer, we make a trade-off between over-fitting the model (too many nodes) and not having enough memory to learn the model (too few nodes). The output of the BiLSTM layer will then be generated based on the output of each separate LSTM layer, as will be discussed in more detail later. It is shown that processing data using BiLSTM layers substantially outperforms unidirectional data processing, i.e., utilizing LSTM layers, due to bidirectional learning long-term dependencies between time steps of time series data [54].

Each LSTM cell is designed to remember information for long periods of time and consists of four neural network layers, as shown in Figure 15. At each time step *t*, LSTM cell has two inputs that are completely different in their function: a cell state (i.e., c_*t*−1_), and a hidden state, (i.e., h_*t*−1_). While a cell state serves as the long-term memory of an LSTM cell and encodes aggregated previously processed data from all previous time steps, the hidden state only encodes the characterization of the previous timestep’s data– also known as working memory that carries information from immediately previous events and overwrites at every step uncontrollably. Each LSTM cell is capable of removing/adding information to the cell state–utilizing two gates: forget gate and input gate. Each gate is formed of a neural network *sigmoid layer σ*. The output of *σ*– which is between zero and one– can be interpreted as the amount of data that can get through or carried forward. While the forget gate decides what information should be discarded from the cell state, the input gate is an aggregate of a *sigmoid layer* and a neural network *tanh layer* which decides what new information needs to be sorted in the new cell state. After deciding what should be removed from or added to the cell state, the updated cell state– in a forward LSTM layer– at the time step *t* can be obtained and written as

(13)
$${\text{c}}_{t}=\overset{\text{Output of the forget gate}}{\overset{︷}{\sigma \left(\omega_{f}{\text{h}}_{t-1}+\theta_{f}x_{t}+\beta_{f}\right)}}⊙{\text{c}}_{t-1}\;+\underset{\text{Output of the input gate}}{\underset{︸}{\sigma \left(\omega_{i}{\text{h}}_{t-1}+\theta_{i}x_{t}+\beta_{i}\right)⊙\text{tanh}\left(\omega_{c}{\text{h}}_{t-1}+\theta_{c}x_{t}+\beta_{c}\right),}}$$

where [*ω*_*f*_, *θ*_*f*_], [*ω*_*i*_, *θ*_*i*_] and [*ω*_*c*_, *θ*_*c*_] denote the forget gate, the input gate, and the candidate cell state weights, respectively, and *β*_*f*_, *β*_*i*_, and *β*_*c*_ represent the bias associated with each layer. Finally, the output gate determines the value of the next hidden state h_*t*_, which is the output of the LSTM cell. The LSTM cell output– in a forward LSTM layer– is a function of current input, current cell state, and previous hidden state:

(14)
$${\text{h}}_{t}=\sigma \left(\omega_{o}{\text{h}}_{t-1}+\theta_{o}x_{t}+\beta_{o}\right)⊙\text{tanh }{\text{c}}_{t},$$

where [*ω*_*o*_, *θ*_*o*_] and *β*_*o*_ denote the output gate weights and the output bias, respectively. In the backward LSTM layer, the output would be calculated based on the reversed input information from the cell state and hidden state. That is, the output is a function of the current input, the current cell state, and the next hidden state. In Figure 14 the output of a LSTM cell in the forward and backward LSTM layers are denoted as $\vec{{\text{h}}_{t}}$ and $\overset{\leftarrow }{{\text{h}}_{t}}$, respectively. These outputs combine to form the output of each cell before being passed on to the next layer:

(15)
$${\text{g}}_{t}=q\left(\vec{{\text{h}}_{t}},\overset{\leftarrow }{{\text{h}}_{t}}\right),$$

which based on the merge mode *q*, the outputs can be either added, multiplied, concatenated, or averaged at each time step. The BiLSTM Layer in our network merges the outputs utilizing the commonly used concatenation mode– which does not discard any inputs.

In this application, after feeding a Wi-Fi CSI data stream to the LSTM network using the sequence input layer, the stream goes through the BiLSTM layer. The output of the BiLSTM layer is an array of 2*N* float numbers which is the outputs of the last *time step* in the Wi-Fi CSI data stream, i.e., **h**_*n*_(*T*), that went through each LSTM layer and concatenated at the end. Note that the number of *time steps* here is the length of the Wi-Fi CSI data stream **h**_*n*_, i.e., *T*. Therefore, the output of the BiLSTM layer– given a Wi-Fi CSI data stream– can be written as

(16)
$$\text{g}={\left[g_{1},\;\ldots \;,g_{2N}\right]}^{T}\in {\mathbb{R}}^{2N}.$$

The concatenated vector **g** then feeds into the next layer.

Based on Eq. (15) and Eq. (16), the output of the BiLSTM layer is a function of the forward and backward hidden states which is not comparable to our respiratory motion-related values. To deal with this issue, we add a fully connected layer on top of the BiLSTM layer. The dimension of this layer is equal to the number of *R* classes that we want to classify in each classification problem, i.e., *R* = 28 for respiratory rates classification, *R* = 3 for broad classes of RRs classification, *R* = 9 for specific respiratory pattern classification, and *R* = 2 for normal vs. abnormal breathing pattern classification as described in more details in Section VI. This layer combines all the information learned by the BiLSTM layer and use them to classify the respiratory motion-related values, i.e., respiratory rate or pattern. To be specific, the fully connected layer constitutes a matrix-vector multiplication:

(17)
u=Wg+b,

where $\text{u}\in {\mathbb{R}}^{R}$ is the output of the fully connected layer, $\text{W}\in {\mathbb{R}}^{R\times 2N}$ is the weight matrix that its elements are a trainable parameter–can get updated during training– and $\text{b}\in {\mathbb{R}}^{R}$ is the bias vector. The elements of output vector **u** are not normalized, so they cannot be displayed to a user to make the final prediction.

The output vector **u** then passes through a softmax layer. This layer converts the output of the fully connected layer into probabilities– each probability assigns to a respiratory motion-related value/class. These probabilities must add up to one– to make training converge more quickly than it otherwise would. The softmax function can be written as follows:

(18)
$$\zeta_{i}(\text{u})=\frac{\text{exp}\left({\text{u}}_{i}\right)}{\sum_{j=1}^{R}\text{exp}\left({\text{u}}_{j}\right)}.$$

Then the output of the softmax layer is vector $\zeta \in {\mathbb{R}}^{R}$ in which each element represents the probability of a list of potential outcomes/classes in each classification problem. Ideally, when we input the Wi-Fi CSI data stream **h**_*n*_ into our network, the network would output an indicator vector of size *R* which the *m*-th element (corresponding to the respiratory motion-related value/class of the Wi-Fi CSI data stream **h**_*n*_) equal to 1, and 0 otherwise. However, the output vector ***ζ*** is not desirable since a perfect network would output a vector of ones and zeros.

To solve this issue, we add one more layer to our network. The last layer of the proposed network is a classification layer that takes the outputs of the softmax layer (***ζ***) and assigns each Wi-Fi CSI data stream (**h**_*n*_) to one of the *R* respiratory pattern/rate classes. As part of the optimization algorithm to do so, the error should be obtained repeatedly and used in the training stage. This necessitates the choice of an error function, also known as the cost/loss function, that can be utilized to estimate the loss of the model. To quantify how far the softmax layer’s output probabilities are from the desired respiratory motion-related values, we apply a cross-entropy loss function, also known as the log-loss function. The loss value can be calculated as

(19)
$$\text{cross-entropy loss }=-\sum_{i=1}^{R}\xi_{i}r_{i}\text{ log}\left(\zeta_{i}\right),$$

where *ξ*_*i*_ and *r*_*i*_ are the weight and the true respiratory rate/motion value for the *i*-th class, respectively. *r*_*i*_ can be also interpreted as the indicator that the input Wi-Fi CSI data stream **h**_*n*_ belongs to the *i*-th class/respiratory motion-related value. The smaller the loss value, the closer the output vector ***ζ*** is to the corresponding correct respiratory motion-related value–a perfect model would have a cross-entropy loss of zero. In the training stage, we then adjust, update and optimize the network’s weights such that the loss value on the next evaluation is reduced– the reduced loss at each iteration indicates that the network has been improved. The optimization algorithm we consider here is the adaptive moment estimation (ADAM) solver to optimize our neural network. The ADAM solver is a common optimizer used in LSTM networks that keeps an element-wise moving average of both the parameter gradients and their squared values. As the loss reduces, the network’s output gets closer to the desired respiratory motion-related values (respiratory rate/pattern), leading to improved CSI-based respiratory monitoring algorithm’s accuracy.
